# Green tea catechin EGCG attenuates hippocampal atrophy and cognitive impairment in obesity via autophagy signaling

**DOI:** 10.1038/s41538-026-00914-4

**Published:** 2026-06-04

**Authors:** Kunyi Huang, Xin Li, Yujie Chen, Yiyun Qi, Weiying Zhang, Jia Ee Tan, Jia Ying Chan, Jiashuo Lu, Fangyi Hao, Jiayi Shi, Shuting Yan, Zhenzhen Huang, Xiaojun Wu, Qi Xu, Zhiwei Ma

**Affiliations:** 1 School of Biomedical Engineering, ShanghaiTech University, Shanghai, China; 2 School of Public Health, Shanghai University of Traditional Chinese Medicine, Shanghai, China; 3 State Key Laboratory of Discovery and Utilization of Functional Components in Traditional Chinese Medicine, Shanghai Key Laboratory of Compound Chinese Medicines, Institute of Chinese Materia Medica, Shanghai University of Traditional Chinese Medicine, Shanghai, China; 4 School of Life Science and Technology, ShanghaiTech University, Shanghai, China; 5 Laboratory Animal Center, Shanghai University of Traditional Chinese Medicine, Shanghai, China; 6 State Key Laboratory of Advanced Medical Materials and Devices, ShanghaiTech University, Shanghai, China; 7 Shanghai Clinical Research and Trial Center, Shanghai, China

**Keywords:** Diseases, Neuroscience

## Abstract

Obesity is prevalent and linked to cognitive impairment via hippocampal atrophy and insulin resistance. Here, we investigated whether the primary catechin of green tea, epigallocatechin‑3‑gallate (EGCG), could attenuate this neuropathology. Epidemiological analysis of UK Biobank adults with obesity provided an initial clue, showing a positive linear trend between green tea intake and hippocampal volume (*p* = 0.07). To elucidate the underlying mechanisms, we administered a human‑achievable dose of EGCG (50 mg/kg) to high‑fat diet-fed mice. EGCG treatment significantly reduced body weight and inflammatory signaling while improving insulin sensitivity, attenuating hippocampal atrophy, and mitigating cognitive deficits. Mechanistically, EGCG rescued synaptic structural integrity by suppressing the pro-inflammatory JNK pathway, restoring hippocampal insulin signaling (IRS1/Akt), and stimulating neuronal autophagy through the AMPK/mTOR/ULK1 axis. Together, these data provide translational evidence that EGCG counteracts obesity-linked neurodegeneration by linking metabolic health to hippocampal integrity through the inflammation–insulin–autophagy axis, motivating dietary trials to mitigate cognitive impairment.

## Introduction

Obesity is a global metabolic disorder characterized by excessive adipose accumulation arising from a multifactorial etiology^1^. Its prevalence has nearly tripled over the past five decades and now represents a major worldwide public health challenge^2^. Concurrently, studies have demonstrated that obesity increases the risk of Alzheimer’s disease (AD), dementia, and cognitive impairment^3^.

Hippocampal atrophy is a sensitive, early biomarker of cognitive dysfunction and a plausible mechanistic bridge between obesity and impaired cognition^4,5^. Structural magnetic resonance imaging (MRI) studies consistently report inverse associations between body mass index (BMI) and hippocampal volume^5,6^. Obesity has further been linked to reduced gray‑matter density and elevated neuron‑specific enolase^7^, and in AD, higher BMI correlates with more pronounced anterior hippocampal atrophy^8^. Preclinical MRI also shows rapid hippocampal alterations after a brief high‑fat diet (HFD) exposure^9^. Consequently, preventing hippocampal atrophy is a compelling target for early intervention in obesity‑related cognitive impairment.

At the molecular level, obesity-induced cognitive impairment is driven by chronic low-grade inflammation and systemic insulin resistance^10^. Adipose tissue expansion promotes the release of pro-inflammatory cytokines, leading to activation of stress kinases such as c-Jun N-terminal kinase (JNK), which in turn disrupts central insulin signaling^11^. Under physiological conditions, insulin is a crucial regulator of neuronal survival and synaptic plasticity, and it plays a key role in various aspects of brain function, including the regulation of food intake, memory, and cognition^12,13^. It acts by binding to insulin receptors to activate downstream signaling pathways such as phosphoinositide 3-kinase (PI3K) / protein kinase B (Akt)^14^. Insulin signaling also plays a pivotal role in modulating cellular homeostasis through the regulation of autophagy—an essential lysosomal degradation pathway that clears damaged organelles and protein aggregates^15^. However, the obesity-induced disruption of brain insulin signaling impairs autophagy activation, and compromises hippocampal synaptic plasticity, thereby accelerating the progression toward cognitive decline^16,17^. Thus, the breakdown of brain insulin signaling constitutes the critical mechanistic link between metabolic obesity and the eventual progression to neurodegeneration.

Beverages in dietary interventions are promising for cognitive preservation due to their high acceptability and minimal disruption to lifestyle^18^. Among these, green tea has gained particular attention due to its antioxidant, anti-inflammatory properties, as well as its ability to promote non-amyloidogenic processing^19^. Several epidemiological investigations have reported an inverse association between green tea consumption and the incidence of age-related cognitive disorders, including mild cognitive impairment (MCI) and dementia^18^. This association was further corroborated by a longitudinal neuroimaging study, which revealed that a higher intake of green tea was associated with a reduced rate of annual atrophy in the hippocampus^20^. However, these neuroprotective effects have not consistently translated into cognitive improvements in clinical trials, prompting a focused investigation into its primary bioactive constituent^21^.

Epigallocatechin-3-Gallate (EGCG) is the most abundant catechin in green tea, representing approximately 59% of the total catechin content^22,23^. Studies have revealed that EGCG exhibits therapeutic effects on cognitive dysfunction, largely due to its capability to stimulate multiple cellular pathways within the brain^23^. Crucially, EGCG and its metabolites can cross the blood-brain barrier (BBB) to reach the brain parenchyma and induce neuritogenesis, thereby enhancing cognitive performance^24^. EGCG is also considered a caloric restriction mimetic (CRM), a class of compounds that confer the health benefits of caloric restriction such as extending lifespan and promoting autophagy without reducing food intake^25^. A brewed cup of green tea contains approximately 200–300 mg of EGCG^26^. Human intervention studies commonly employ EGCG doses ranging from 150 to 800 mg/day, which can be achieved through concentrated extracts^27,28^. In preclinical translational studies, mice are frequently administered 50 mg/kg/day EGCG, a dose equivalent to 4.1 mg/kg/day in adults and is both effective and safe for glucose metabolism in obese individuals^28–30^, enabling direct investigation of EGCG-specific mechanisms of action.

In the present study, we investigated whether green tea—and specifically its main bioactive constituent EGCG—attenuates hippocampal atrophy and cognitive impairment in obesity by modulating the inflammation-insulin-autophagy pathway. First, using UK Biobank imaging data, we examined whether green tea intake relates to hippocampal volume in adults with obesity. We then validated putative mechanisms in HFD‑fed mice using a human‑achievable EGCG dose (50 mg/kg), combining longitudinal metabolic phenotyping, MRI‑based volumetry, and behavioral assays. Finally, we interrogated synaptic integrity (PSD95) alongside upstream hippocampal inflammation (p-JNK), insulin signaling (IRS1/Akt), and autophagy pathways (AMPK–SIRT1 and AMPK–mTOR–ULK1) to link systemic metabolic health with brain structure and function.

## Results

To investigate whether green tea and its principal catechin, EGCG, attenuate obesity-linked hippocampal atrophy and cognitive impairment, we employed a translational approach integrating human observational data with mechanistic animal experiments. We first analyzed UK Biobank brain MRI and dietary data to evaluate whether green tea intake is associated with hippocampal volume in adults with obesity. We then tested causality and underlying mechanisms in high-fat diet-fed mice treated with a human-achievable dose of EGCG, assessing metabolic health, hippocampal structure, cognitive performance, synaptic integrity, and the inflammation–insulin–autophagy signaling axis.

### Green tea consumption shows a positive trend with hippocampal volume in adults with obesity

**Fig. 1:**
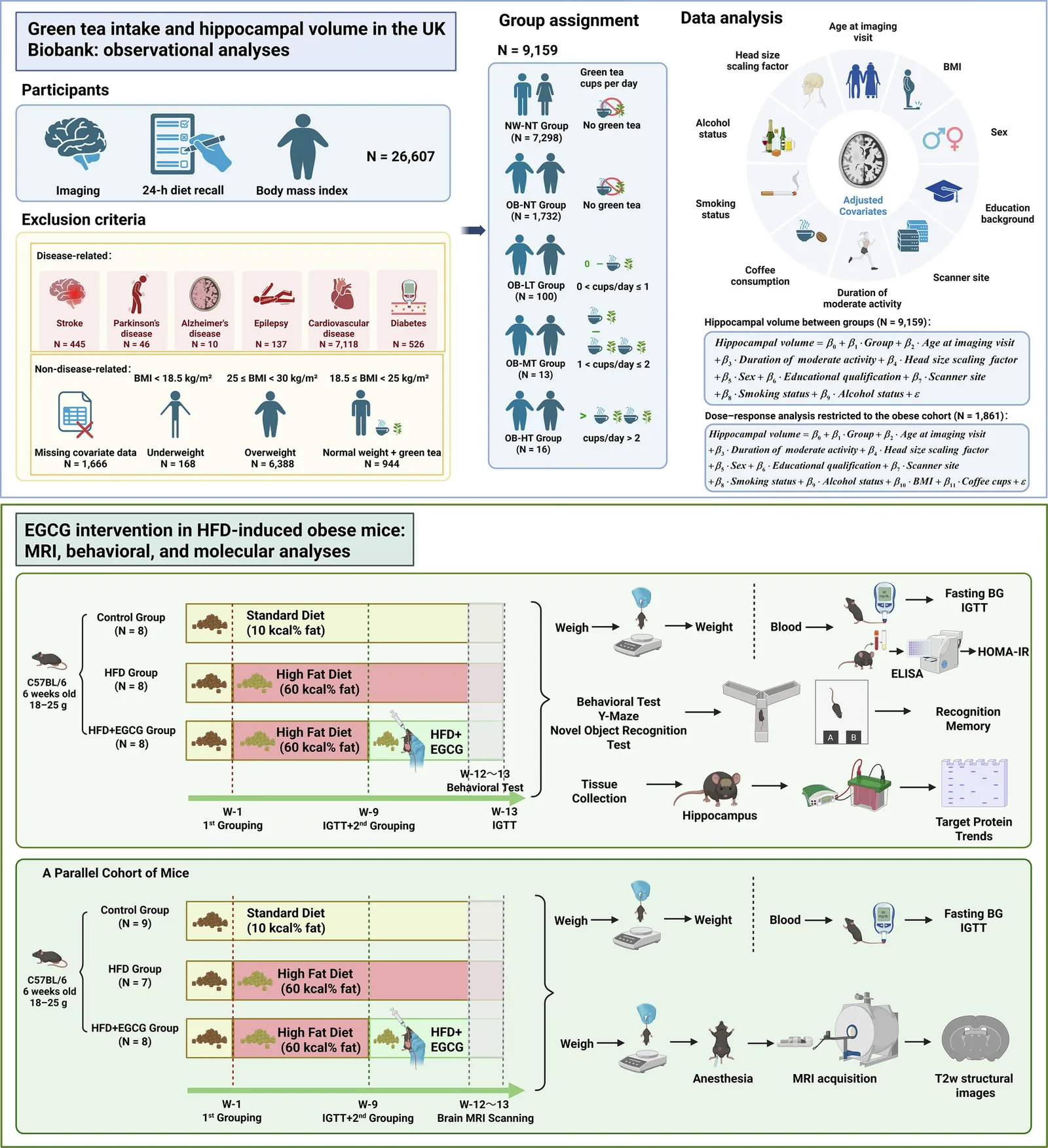
Study overview. The study integrates a population‑based analysis of the UK Biobank with mechanistic validation in high-fat diet (HFD)‑fed mice to test whether green tea intake is associated with hippocampal volume, and whether epigallocatechin-3-gallate (EGCG) attenuates hippocampal atrophy and cognitive impairments in obesity via autophagy signaling. Created in BioRender. Ma, Z. (2026) https://BioRender.com/803p4f6. BMI Body mass index, NW-NT Normal weight with no tea, OB-MT Obese with moderate green tea intake, OB-HT Obese with high green tea intake, EGCG Epigallocatechin-3-Gallate, HFD High-fat diet, HFD + EGCG High-fat diet combined with epigallocatechin-3-gallate, BG Blood glucose, IGTT Intraperitoneal glucose tolerance test, ELISA Enzyme-linked immunosorbent assay, HOMA-IR Homeostasis model assessment of insulin resistance.

**Fig. 2:**
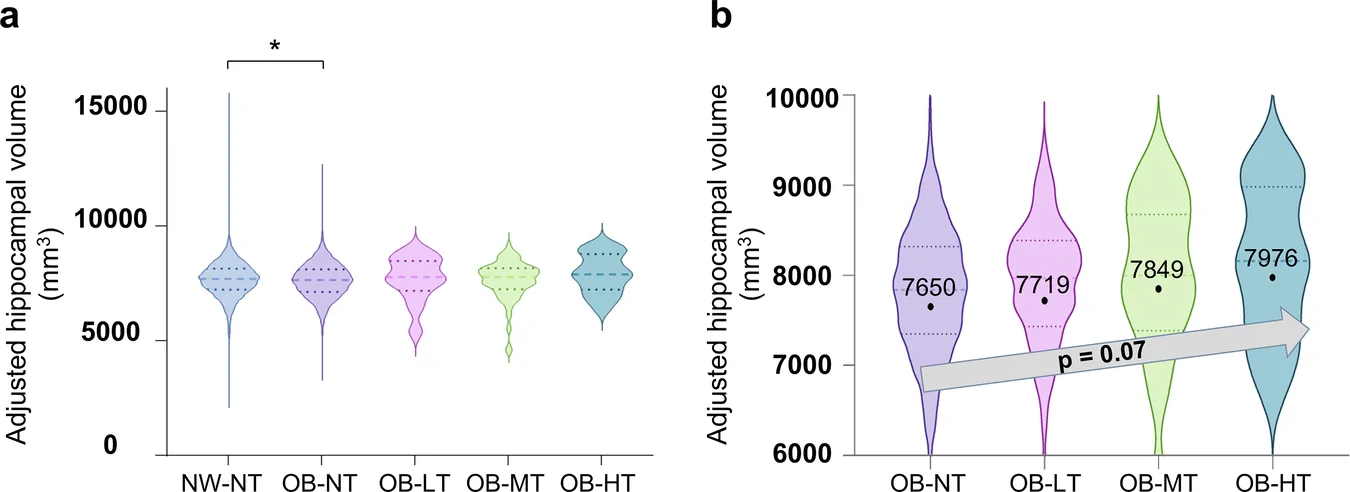
Association between green tea intake and hippocampal volume. **a** Hippocampal volume across groups. Post‑hoc pairwise comparison showed smaller hippocampal volume in OB‑NT versus NW‑NT (*p* = 0.04; *p < 0.05). **b** Dose–response analysis restricted to individuals with obesity (OB-NT, OB-LT, OB-MT, and OB-HT). Compared to (**a**), the model in (**b**) includes BMI and coffee consumption as covariates. The numerical values above the black dots in (**b**) denote Estimated Marginal Means (EMMs), and the grey arrow indicates a positive dose-response trend (*p* = 0.07). Violin plots represent the distribution density, with dashed and dotted lines indicating the median and interquartile ranges, respectively. NW-NT Normal weight and no tea intake, OB-NT Obese and no tea intake, OB-LT Obese and low green tea intake, OB-MT Obese and moderate green tea intake, OB-HT Obese and high green tea intake.

Using data from the UK Biobank, we examined the association between obesity, green tea intake, and hippocampal volume (Fig. 1). Specifically, we analyzed structural MRI and dietary data from participants free of neurological or cardiovascular disease. They were stratified into a normal-weight non-tea reference group (NW-NT; *n* = 7,298) and four obese subgroups based on daily green tea consumption: no tea (OB-NT; *n* = 1,732), low tea (OB-LT; *n* = 100), moderate tea (OB-MT; *n* = 13), and high tea (OB-HT; *n* = 16). The demographics and health characteristics of the participants are shown in Table S1. Post-hoc analysis revealed a significantly smaller hippocampal volume in the OB-NT group compared to the NW-NT group (*p* < 0.05, Fig. 2a), suggesting an adverse effect of obesity on hippocampal integrity. No other significant differences in hippocampal volume were observed between any other groups (*p* > 0.05, Table S2).

Within individuals with obesity, we further analyzed estimated marginal means (EMMs) of hippocampal volume across levels of green tea intake, after adjusting for BMI and coffee consumption alongside all other baseline covariates. The adjusted EMMs indicated a graded increase in hippocampal volume with higher tea intake, rising from 7650 mm³ (95% CI 7416–7884) in OB‑NT to 7976 mm³ (95% CI 7540–8411) in the OB‑HT group (Table S3). Polynomial trend analysis revealed a trend-level positive linear association between tea intake and hippocampal volume (*p* = 0.07, Fig. 2b). To evaluate the robustness of this trend given the unequal group sizes, we performed a stratified bootstrap analysis (2000 resamples), which confirmed that 88.4% of iterations yielded a positive linear slope, indicating the directional stability of the trend. In a complementary sensitivity analysis that collapsed all obese tea consumers into a single group (OB-Tea), bootstrap resampling similarly showed a consistent positive volumetric difference against the OB-NT group (92.2% positive iterations; median increase 106.4 mm³). This population-based analysis provides a rationale for investigating whether green tea components might enhance neuroprotective effects in the hippocampus in the context of obesity.

### EGCG reduces body weight gain and improves glucose tolerance in HFD-fed mice

To investigate the mechanisms underlying our population-level observations, we evaluated the protective effects of EGCG, the predominant catechin in green tea, against HFD-induced metabolic and neuropathological changes in a mouse model of obesity. Male C57BL/6 mice were fed either a standard chow diet (Control, *n* = 8) or a high-fat diet (HFD, *n* = 16; 60 kcal% fat) for 8 weeks to induce obesity. HFD-fed mice were then randomized to continue on the HFD alone (*n* = 8) or to receive daily oral EGCG (50 mg/kg body weight; HFD + EGCG, *n* = 8) for an additional 4 weeks. This dose corresponds to approximately 4.1 mg/kg/day in humans by body surface area scaling and is achievable through dietary supplementation (see *Methods* for details).

**Fig. 3:**
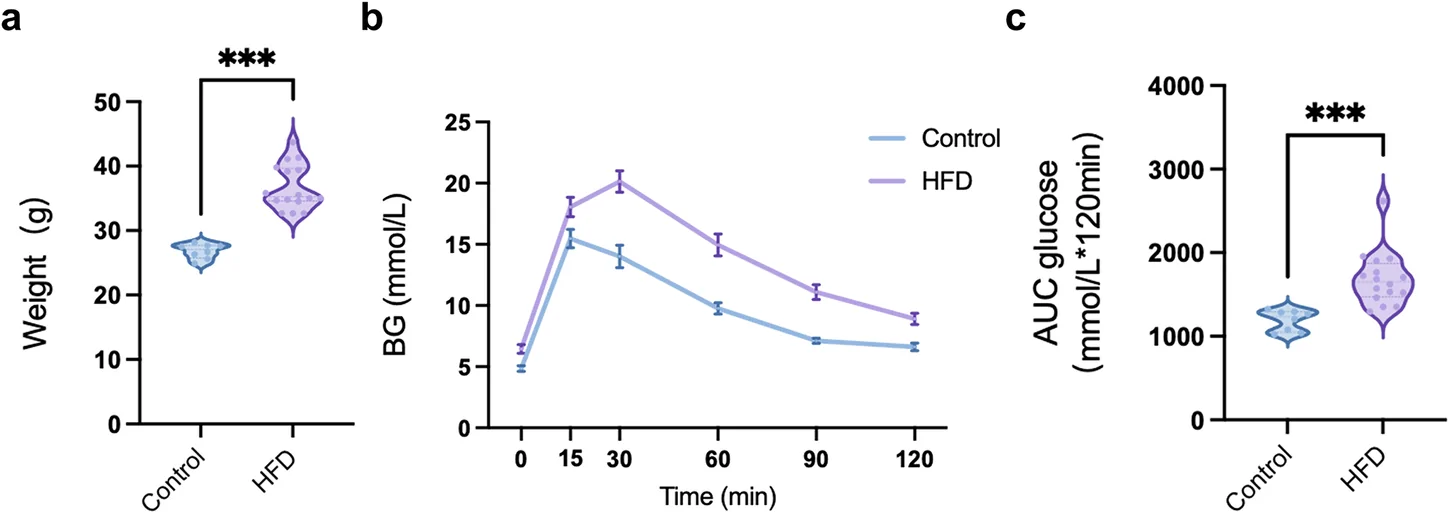
Effects of an HFD on body weight and insulin resistance at week 8. **a** Body weight (Welch’s t‑test; Control: *n* = 8; HFD: *n* = 16). Within the violin plots, dark and light dashed lines represent the median and interquartile ranges, respectively (standardized across subsequent violin plots unless noted otherwise). **b** IGTT time course (0–120 min; Control: *n* = 8; HFD: *n* = 16). **c** IGTT area under the curve (AUC) (Mann–Whitney U test; Control: *n* = 8; HFD: *n* = 16). Data are mean±standard error of the mean (SEM). Comparisons are versus the Control. ***p < 0.001. IGTT Intraperitoneal glucose tolerance test, HFD High-fat diet, BG Blood glucose, AUC Area under the curve, SEM Standard error of the mean.

At the baseline, body weight did not differ significantly between the Control and HFD groups (Unpaired t‑test; Fig. S2a). Over the initial 8-week period, body weight progression showed a clear divergence between the groups following diet initiation (Fig. S2b). By week 8, the HFD group exhibited a 37% increase in body weight and a 41.9% increase in glucose tolerance AUC compared to the Control group (*p* < 0.001; Welch’s t‑test for body weight and Mann–Whitney U test for AUC; Fig. 3a, c), indicating HFD-induced weight gain and glucose intolerance.

**Fig. 4:**
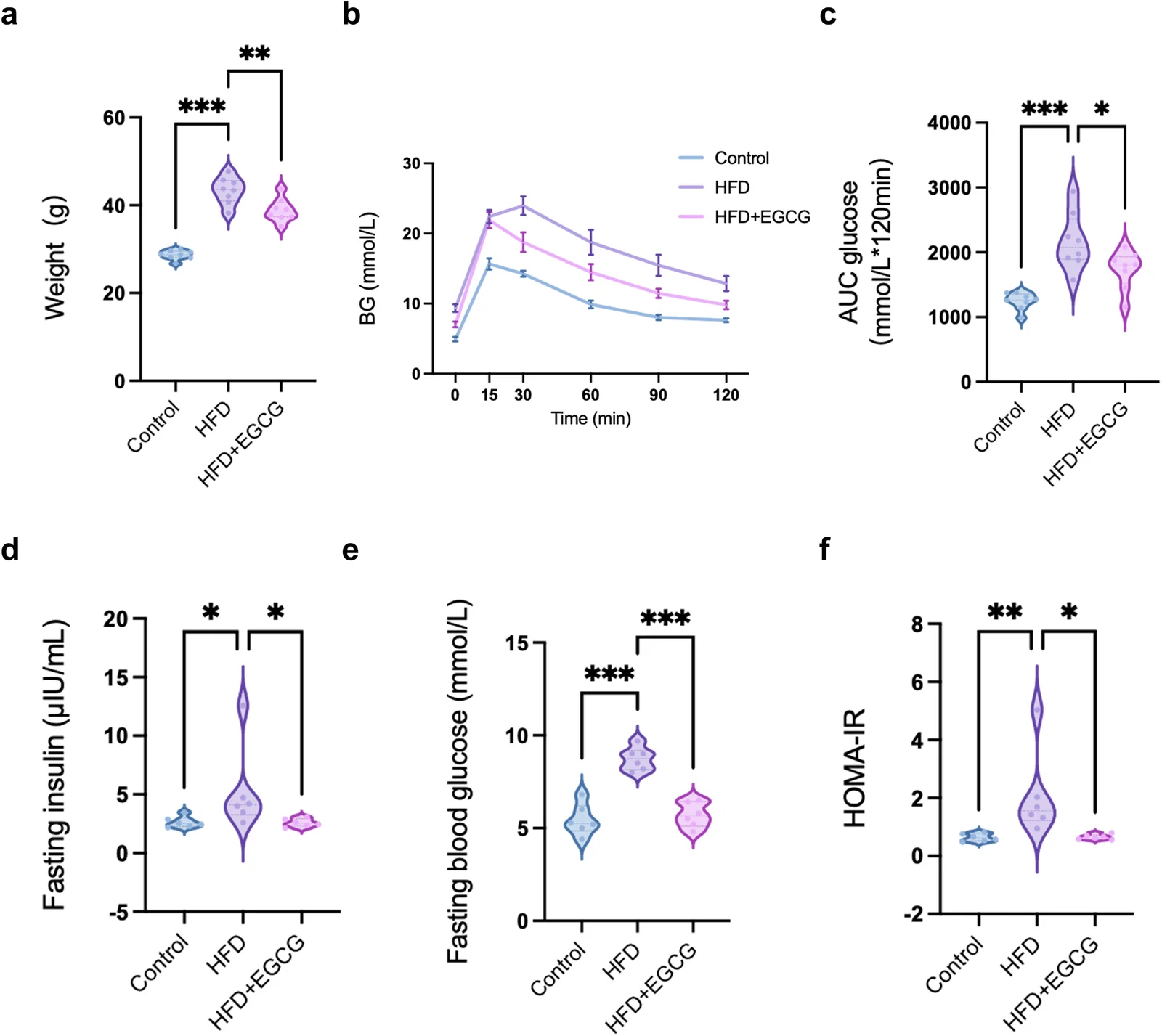
EGCG attenuates HFD‑induced weight gain and insulin resistance. **a** Body weight (one‑way ANOVA with Dunnett’s multiple comparisons test; *n* = 8/group). **b** IGTT time course (0–120 min; *n* = 8/group). **c** IGTT AUC (one‑way ANOVA with Dunnett’s multiple comparisons test; *n* = 8/group). **d** Fasting insulin (Kruskal–Wallis test with Dunn’s multiple comparisons test; *n* = 6/group). **e** Fasting blood glucose (one‑way ANOVA with Dunnett’s multiple comparisons test; *n* = 6/group). **f** Homeostasis model assessment of insulin resistance (HOMA‑IR) (Kruskal–Wallis test with Dunn’s multiple comparisons test; *n* = 6/group). Data are mean±SEM. Comparisons are versus the HFD group. *p < 0.05, **p < 0.01, ***p < 0.001. EGCG Epigallocatechin-3-Gallate, HFD High-fat diet, HFD + EGCG High-fat diet combined with epigallocatechin-3-gallate, BG Blood glucose, IGTT Intraperitoneal glucose tolerance test, HOMA-IR Homeostasis model assessment of insulin resistance, SEM Standard error of the mean.

Following the 4-week intervention period, EGCG treatment significantly reduced body weight by 10.3% (*p* < 0.01; Fig. 4a) and AUC by 20.0% (*p* < 0.05; Fig. 4c) compared to the HFD group (one‑way ANOVA with Dunnett’s post hoc test). To further assess insulin sensitivity, we measured fasting insulin and glucose levels after four weeks of EGCG. EGCG treatment markedly attenuated these elevations, reducing fasting insulin by 50.9% (*p* < 0.05; Fig. 4d) and glucose levels by 34.7% (*p* < 0.001; Fig. 4e) relative to the HFD group (Kruskal–Wallis test with Dunn’s multiple‑comparisons test for fasting insulin and one‑way ANOVA with Dunnett’s post hoc test for fasting glucose). Consequently, the homeostasis model assessment of insulin resistance (HOMA-IR) index was decreased by 68.6% in the EGCG group compared to the HFD group (*p* < 0.05; Kruskal–Wallis test with Dunn’s multiple‑comparisons test; Fig. 4f). Collectively, these data demonstrate that EGCG improves systemic insulin sensitivity by concurrently mitigating HFD‑induced high insulin and hyperglycemia.

### EGCG attenuates HFD-induced hippocampal atrophy

**Fig. 5:**
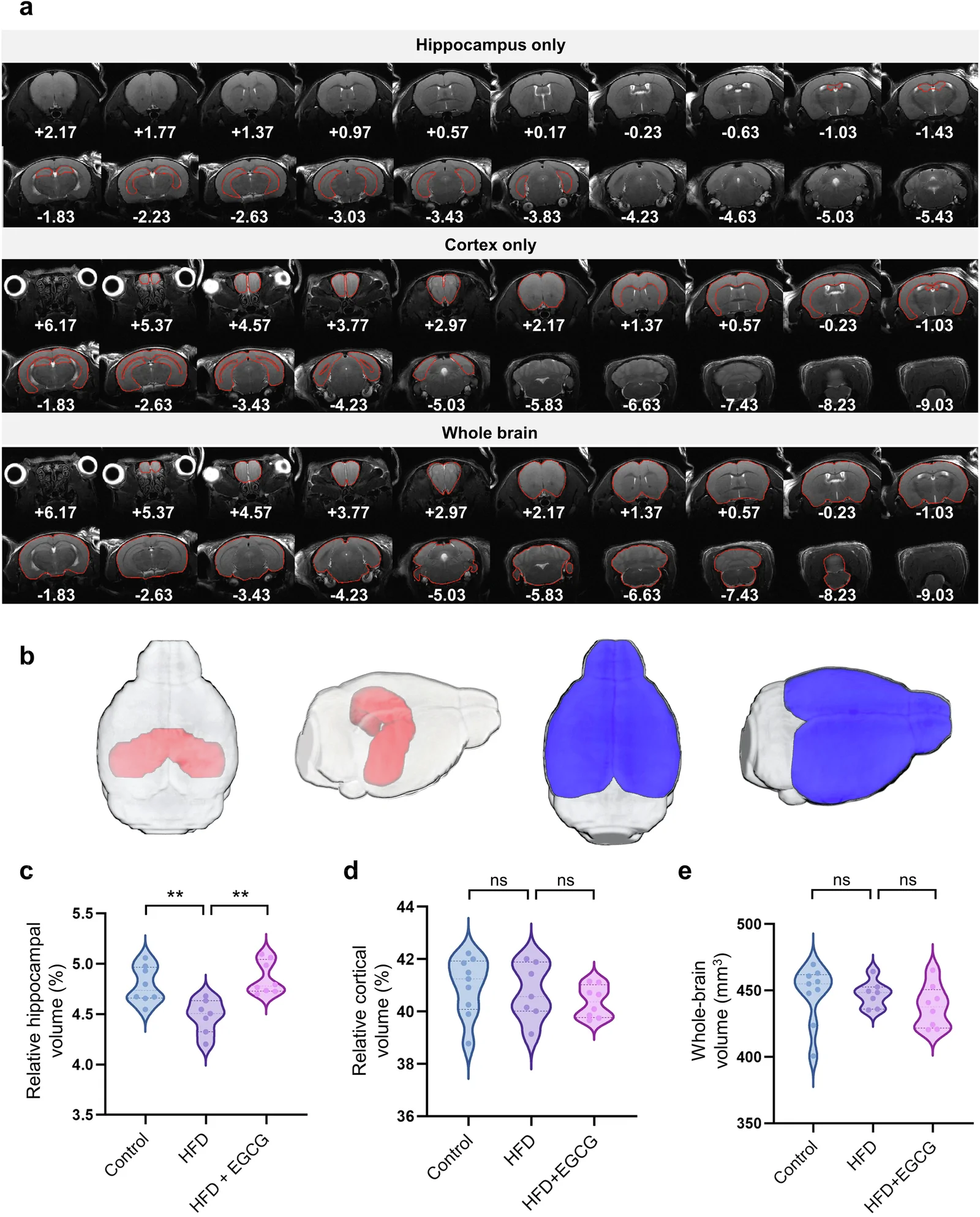
EGCG attenuates HFD-induced hippocampal atrophy without altering cortical or whole‑brain volumes. **a** Representative manual segmentations of the hippocampus, cerebral cortex, and whole brain, demonstrating precise anatomical delineation based on key landmarks including the lateral ventricles, cortical surface, and corpus callosum. For visualization, MRI structural slices of the hippocampus are displayed contiguously, whereas slices of the cerebral cortex and whole brain are presented at every other slice to demonstrate anatomical delineation. **b** Three-dimensional reconstruction of the hippocampus and cortex in the mouse brain, illustrating comprehensive volumetric coverage. The hippocampus (red) is visualized within a transparent whole-brain surface, and the cerebral cortex (blue) is rendered as a surface overlay. Both structures are shown from dorsal and lateral perspectives. **c** Relative hippocampal volume (one‑way ANOVA with Bonferroni’s multiple comparisons test; *n* = 7–9/group). **d** Relative cortical volume (one‑way ANOVA with Dunnett’s multiple comparisons test; *n* = 7–9/group). **e** Whole‑brain volume (Kruskal–Wallis test with Dunn’s multiple comparisons test; *n* = 7–9/group). Data are mean±SEM. Comparisons are versus the HFD group. **p < 0.01. EGCG Epigallocatechin-3-Gallate, HFD High-fat diet, HFD + EGCG High-fat diet combined with epigallocatechin-3-gallate, SEM Standard error of the mean.

A parallel cohort (Control, *n* = 9; HFD, *n* = 7; HFD + EGCG, *n* = 8) underwent identical dietary modeling and was dedicated to structural MRI. To evaluate the effects of HFD and EGCG supplementation on hippocampal structure, we compared relative hippocampal volume across the three experimental groups. Representative manual segmentations of the hippocampus, cerebral cortex, and whole brain are shown in Fig. 5a, alongside a corresponding 3D rendering in Fig. 5b. Consistent with the human data, relative hippocampal volume was significantly reduced in the HFD group compared to Control: the HFD group exhibited a 6.3% reduction in relative hippocampal volume compared to the Control group (*p* < 0.01, one-way ANOVA followed by Bonferroni’s multiple comparison test, Fig. 5c). Notably, EGCG treatment reversed this deficit; the HFD + EGCG group exhibited an 8.5% increase in relative hippocampal volume compared to the HFD group (*p* < 0.01), suggesting a restoration of hippocampal volume. No significant differences were found in relative cortical volume (*p* > 0.05, one-way ANOVA with Dunnett’s multiple comparisons test, Fig. 5d) or whole brain volume (Kruskal-Wallis test followed by Dunn’s multiple comparisons test, Fig. 5e) among the three groups. These results indicate that an HFD may selectively reduce hippocampal volume, whereas EGCG supplementation mitigates this effect, preserving hippocampal integrity without altering global brain or cortical morphology.

### EGCG ameliorates HFD-induced cognitive impairments

**Fig. 6:**
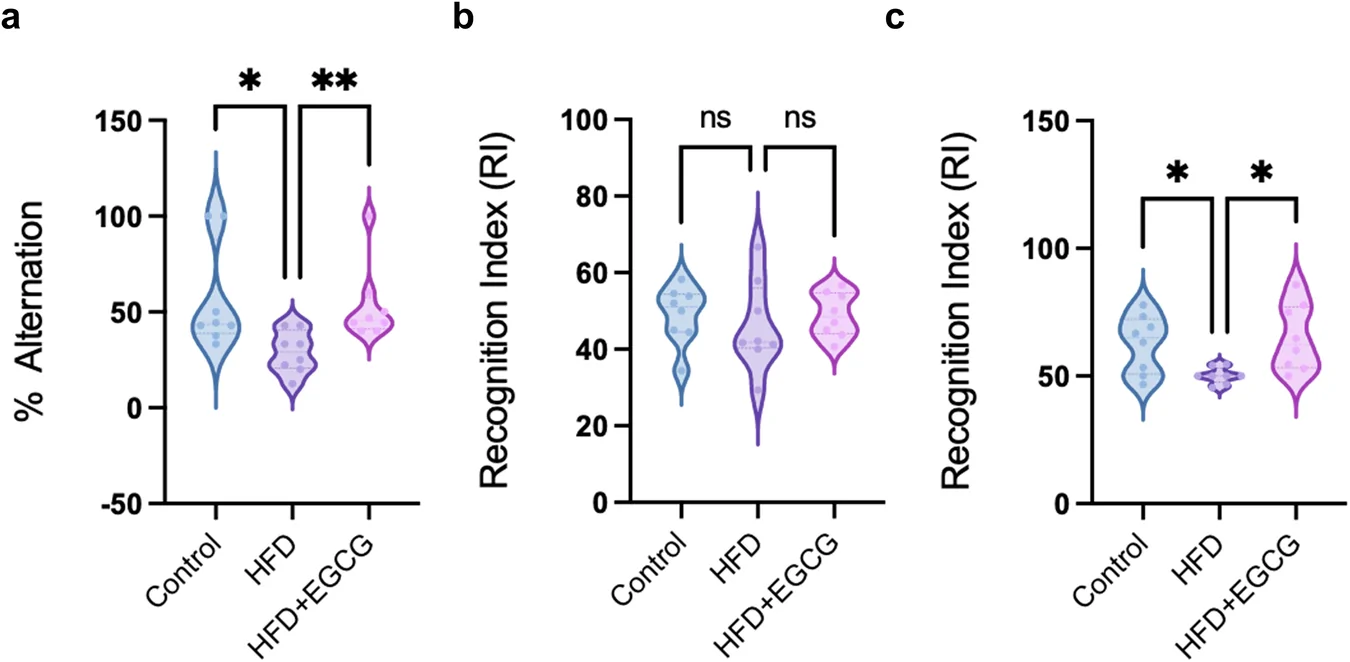
EGCG rescues HFD‑induced memory deficits. **a** Y‑maze spontaneous alternation (Kruskal–Wallis test with Dunn’s multiple comparisons test; *n* = 8/group). **b** Novel object recognition (NOR) recognition index (acquisition) (one‑way ANOVA with Dunnett’s multiple comparisons test; *n* = 8/group). (**c**) NOR recognition index (test) (Brown–Forsythe and Welch ANOVA with Dunnett’s T3 multiple comparisons test; *n* = 8/group). Data are mean±SEM. Comparisons are versus the HFD group. *p < 0.05, **p < 0.01. EGCG Epigallocatechin-3-Gallate, HFD High-fat diet, HFD + EGCG High-fat diet combined with epigallocatechin-3-gallate, NOR Novel object recognition test, RI Recognition index, SEM Standard error of the mean.

We evaluated changes in learning and memory in obese mice and the beneficial effects of EGCG using the Y-maze and novel object recognition (NOR) tests. In the Y-maze, the frequency of spontaneous alternations was 48.5% lower in the HFD group compared to the Control group (*p* < 0.05, Kruskal-Wallis test with Dunn’s multiple comparisons test, Fig. 6a). This reduction was significantly counteracted by EGCG treatment, resulting in an 83.3% increase relative to the HFD group (*p* < 0.01).

In the NOR test, a One-sample t-test confirmed no side bias during the acquisition phase, as the investigation ratio for each group did not differ from the 50% chance level (Control: 49.07 ± 2.68%; HFD: 46.12 ± 4.13%; HFD + EGCG: 49.01 ± 2.05%; *p* > 0.05, One-sample *t*-test; Table S4). Moreover, no significant differences in RI were detected among groups during the training (*p* > 0.05, one‑way ANOVA with Dunnett’s post hoc test, Fig. 6b). During the test phase, both the Control and HFD + EGCG groups displayed a significant preference for the novel object, with discrimination indices exceeding chance (Control: 62.52 ± 4.02%, *p* < 0.05; HFD + EGCG: 65.09 ± 4.62%, *p* < 0.05; One-sample t-test, Table S5). In contrast, the HFD group did not deviate from chance (50.10 ± 1.12%, *p* > 0.05, One-sample t-test). Consistent with this, inter‑group comparisons revealed a 19.9% reduction in the recognition index of the HFD group (*p* < 0.05, Brown-Forsythe and Welch ANOVA test with Dunnett T3 multiple comparisons test, Fig. 6c). This impairment was fully rescued by four-week EGCG administration, which restored RI by 29.9% relative to the HFD group (*p* < 0.05).

To exclude the confounding effects of motor activity or anxiety on cognitive performance, we also performed the open field test (OFT). No significant differences were found among the groups in total distance traveled or time spent in the center zone (one‑way ANOVA with Dunnett’s post hoc test for total distance traveled and Kruskal-Wallis test with Dunn’s multiple comparisons test for the time spent in the center, Fig. S3).

### EGCG mitigated HFD-induced neuroplasticity dysfunction

**Fig. 7:**
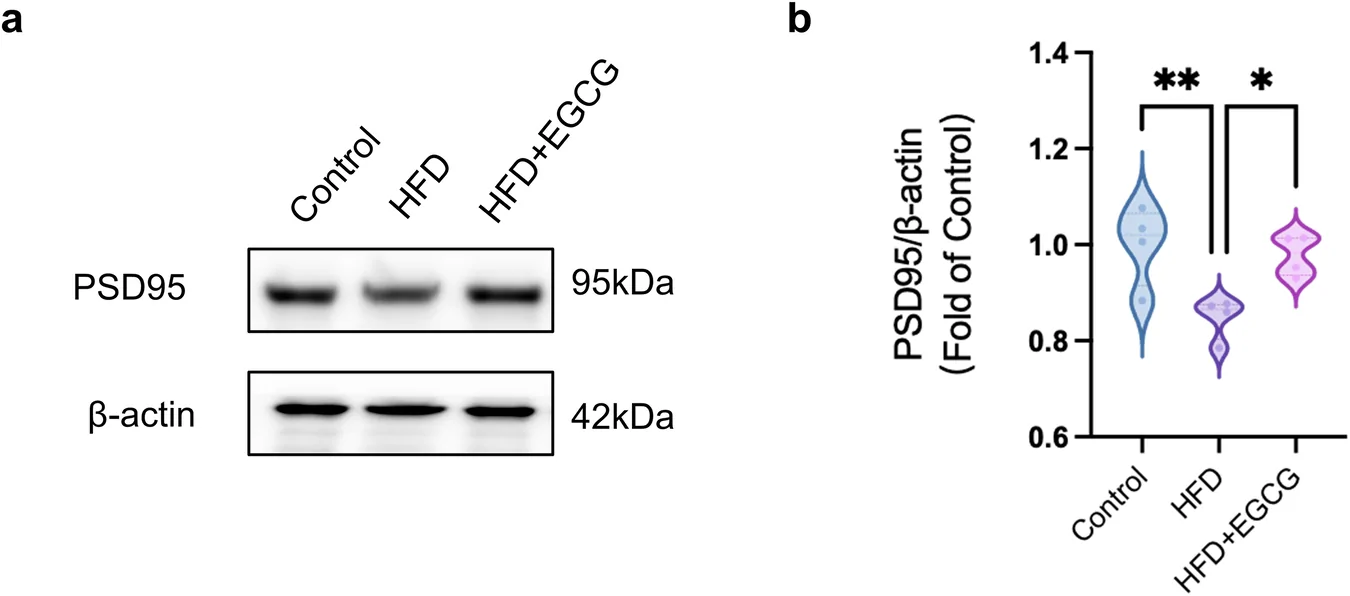
EGCG restores the stability of hippocampal synaptic structures damaged by HFD. **a** Representative immunoblots. **b** Densitometry of synaptic integrity‑related proteins in hippocampus (one‑way ANOVA with Dunnett’s multiple comparisons test; *n* = 4/group), normalized to β‑actin. Data are mean±SEM. Comparisons are versus the HFD group. *p < 0.05, **p < 0.01. EGCG Epigallocatechin-3-Gallate, HFD High-fat diet, HFD + EGCG High-fat diet combined with epigallocatechin-3-gallate, SEM Standard error of the mean.

To investigate the cellular basis of the observed cognitive impairments, we evaluated the expression of the mature neuronal marker NeuN (Fig. S4) and the postsynaptic protein PSD95 (Fig. 7a) in the hippocampus. The NeuN^+^ cell density was significantly reduced in the hippocampal CA3 region of HFD-fed mice (*p* < 0.05; one-way ANOVA with Dunnett’s test, Fig. S4b). Moreover, PSD95 protein levels were decreased by 15.2% compared to the Control group (*p* < 0.01; one-way ANOVA with Dunnett’s test, Fig. 7b). In contrast, EGCG intervention effectively counteracted these alterations, preventing the loss of NeuN+ neurons (*p* < 0.05, Fig. S4b) and restoring PSD95 expression to control levels (*p* < 0.05, Fig. 7b). These results demonstrate that EGCG protects against HFD-induced neuronal damage and synaptic dysfunction.

### EGCG inhibits JNK activation and restores hippocampal insulin signaling in HFD-fed mice

**Fig. 8:**
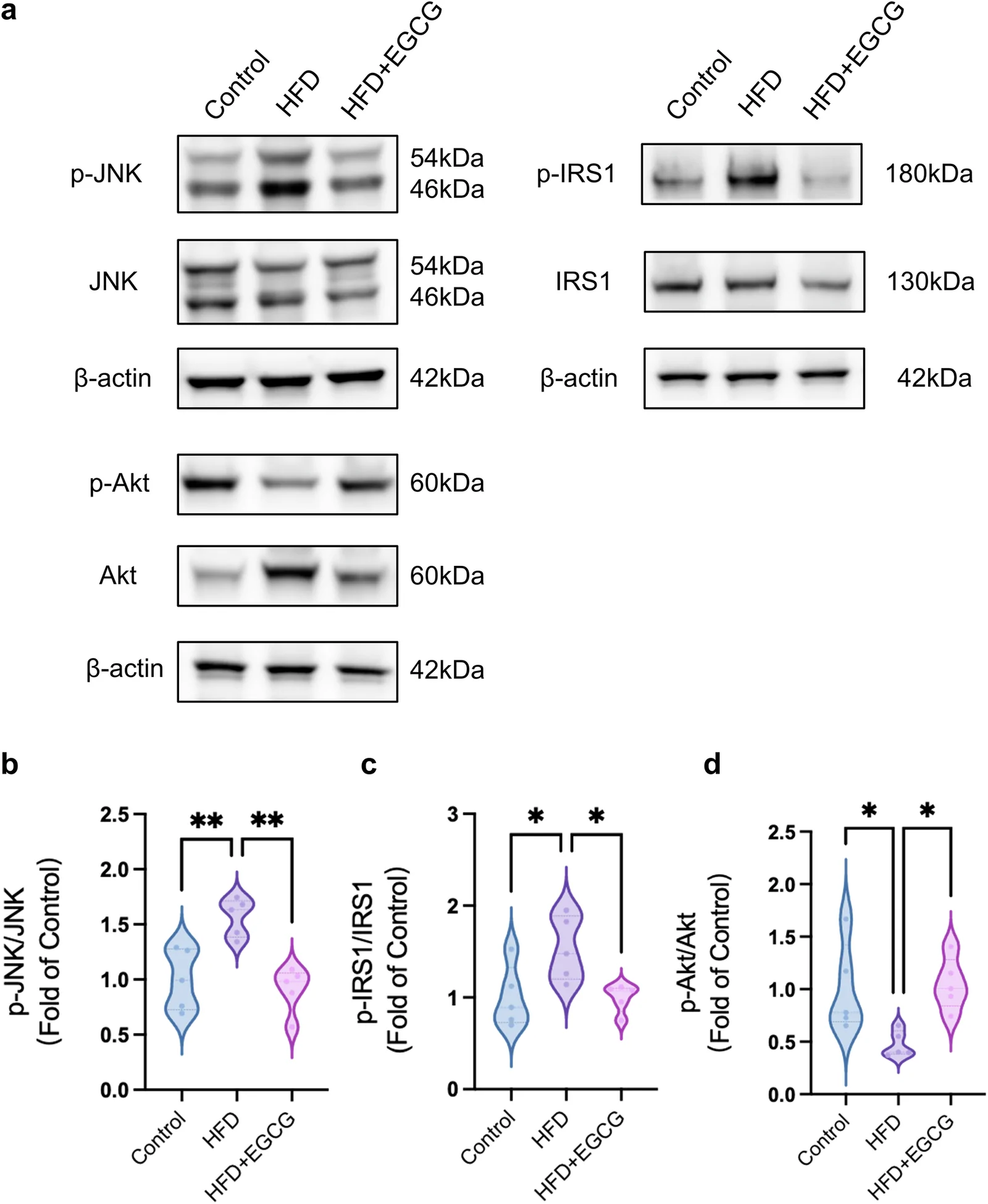
EGCG reduces inflammation and reverses HFD‑induced impairments in hippocampal insulin signaling. **a** Representative immunoblots. **b** Densitometry of inflammatory proteins in hippocampus. **c**, **d** Densitometry of insulin‑signaling proteins in hippocampus (one‑way ANOVA with Dunnett’s multiple comparisons test; *n* = 5/group), normalized to β‑actin. Data are mean±SEM. Comparisons are versus the HFD group. *p < 0.05, **p < 0.01. EGCG Epigallocatechin-3-Gallate, HFD High-fat diet, HFD + EGCG High-fat diet combined with epigallocatechin-3-gallate, SEM Standard error of the mean.

We next examined the impact of EGCG on hippocampal inflammatory signaling as indicated by JNK and insulin signaling pathways (Fig. 8a). Relative to the Control group, HFD-fed mice exhibited a 56.6% increase in the phosphorylation of JNK (*p* < 0.01) and a 53.0% increase in the inhibitory phosphorylation of IRS1 at Ser307 (*p* < 0.05). Concurrently, Akt phosphorylation was reduced by 52.5% (*p* < 0.05) in HFD mice (one‑way ANOVA with Dunnett’s multiple‑comparisons; Fig. 8b–d). EGCG partially reversed these effects, reducing JNK phosphorylation by 41.8% (*p* < 0.01) and IRS1‑Ser307 phosphorylation by 34.9% (*p* < 0.05). Furthermore, EGCG treatment led to a 1.2-fold increase in Akt phosphorylation compared to the HFD group (*p* < 0.05). Collectively, these results indicate that EGCG suppresses HFD-induced inflammatory signaling and restores essential insulin signaling cascades within the hippocampus.

### EGCG activates hippocampal autophagy associated with AMPK-dependent pathways

**Fig. 9:**
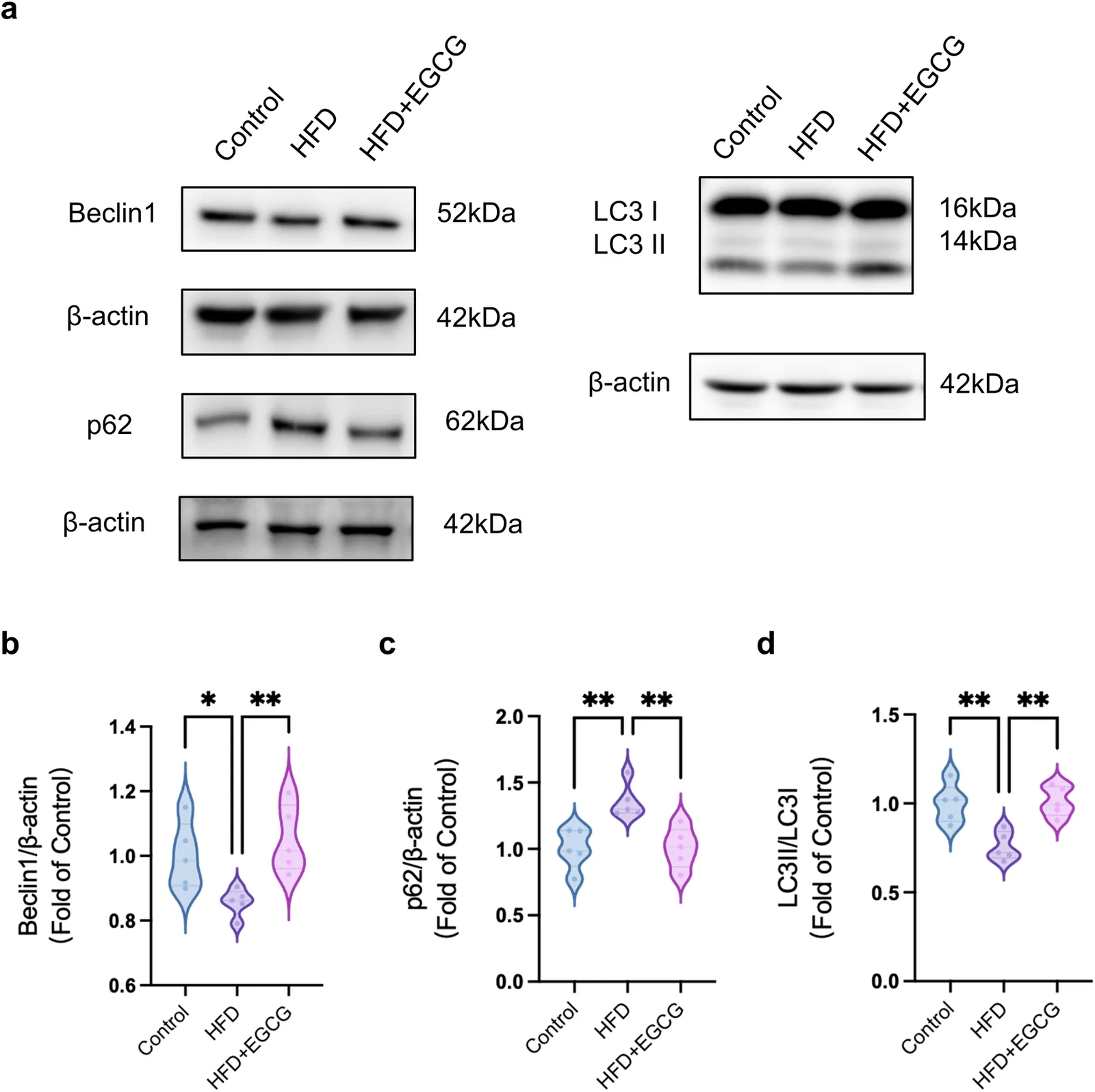
EGCG enhances hippocampal autophagy markers. **a** Representative immunoblots. **b**-**d** Densitometry of autophagy‑related proteins in hippocampus (one‑way ANOVA with Dunnett’s multiple comparisons test; *n* = 5/group), normalized to β‑actin. Data are mean±SEM. Comparisons are versus the HFD group. *p < 0.05, **p < 0.01. EGCG Epigallocatechin-3-Gallate, HFD High-fat diet, HFD + EGCG High-fat diet combined with epigallocatechin-3-gallate, SEM Standard error of the mean.

To investigate the downstream neuroprotective mechanism of EGCG in obese mice, we assessed the expression of autophagy-related proteins within the hippocampus (Fig. 9a). Compared to the Control group, the HFD group exhibited a 14.3% decrease (*p* < 0.05) in Beclin1 expression and a 24.1% reduction in the LC3‑II/LC3‑I ratio (*p* < 0.01). These changes were accompanied by a significant 35.8% (*p* < 0.01) increase in p62 (One-way ANOVA with Dunnett’s multiple comparisons test, Fig. 9b–d), indicating impaired autophagic clearance. EGCG treatment activated the autophagic signaling pathway, increasing Beclin1 and the LC3‑II/LC3‑I ratio by 22.6% and 33.2% (*p* < 0.01), respectively, compared to the HFD group. Furthermore, EGCG downregulated p62 by 25.7% (*p* < 0.01), confirming restored autophagic flux. These biochemical findings were further supported by representative ultrastructural observations of the hippocampal CA3 region via transmission electron microscopy (TEM). Consistent with the protein expression data, hippocampal tissue from HFD-fed mice showed fewer identifiable autophagosomes and autolysosomes than tissue from the Control group, whereas tissue from EGCG-treated mice displayed a noticeable presence of autophagic structures (Fig. S5).

**Fig. 10:**
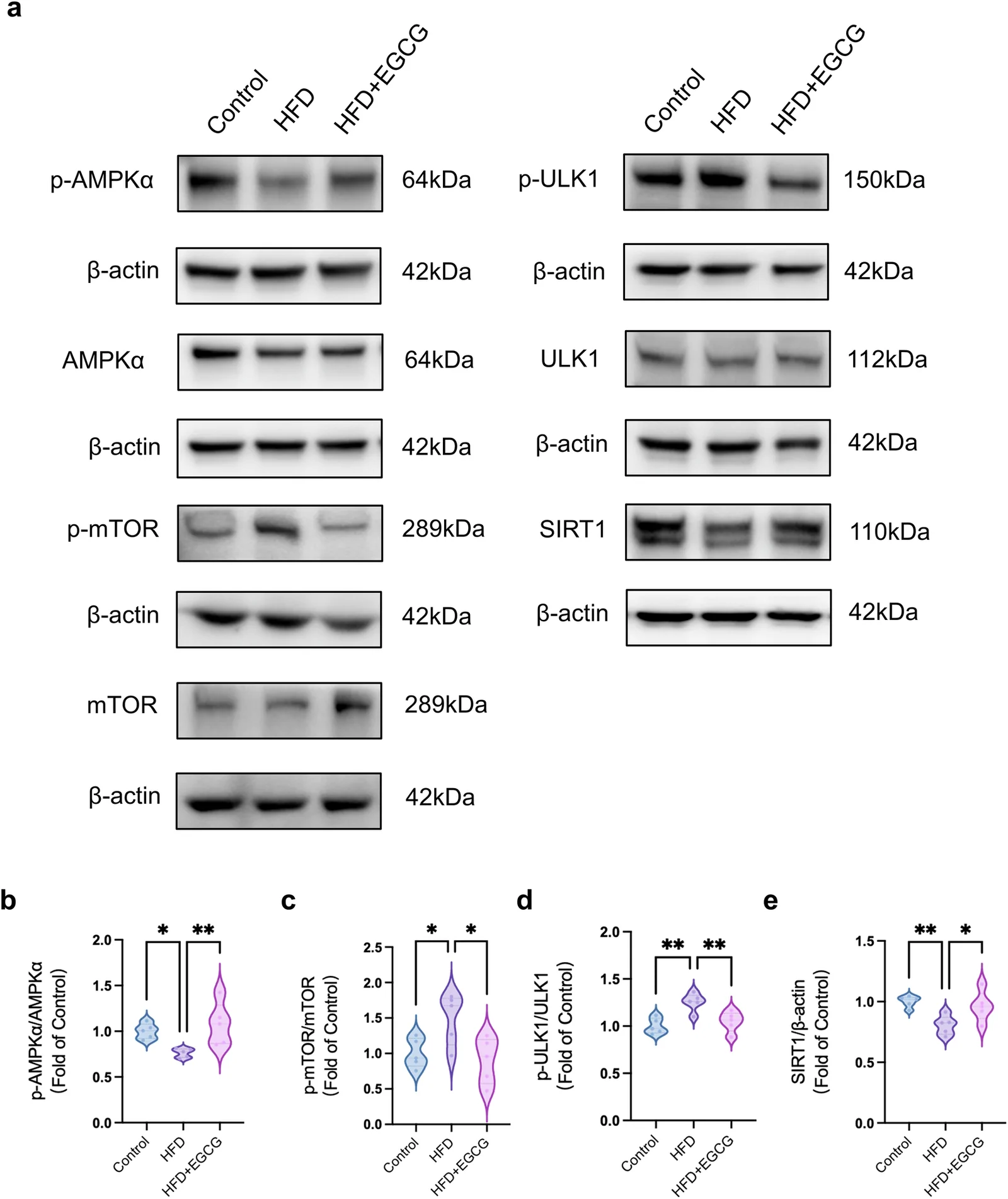
EGCG activates hippocampal autophagy via AMPK–SIRT1 and AMPK–mTOR–ULK1 signaling. **a** Representative immunoblots. **b**–**e** Densitometry of pathway proteins in hippocampus (one‑way ANOVA with Dunnett’s multiple comparisons test; *n* = 5/group), normalized to β‑actin. Data are mean±SEM. Comparisons are versus the HFD group. *p < 0.05, **p < 0.01. EGCG Epigallocatechin-3-Gallate, HFD High-fat diet, HFD + EGCG High-fat diet combined with epigallocatechin-3-gallate, AMPK AMP-activated protein kinase, mTOR Mechanistic target of rapamycin, ULK1 Unc-51 like autophagy activating kinase 1, SIRT1 Sirtuin 1, SEM Standard error of the mean.

To further examine the mechanisms by which EGCG induced autophagy, we investigated the proteins involved in the AMPK/SIRT1 and AMPK/mTOR/ULK1 pathways (Fig. 10a). Our findings revealed that HFD consumption suppressed the AMPK/mTOR/ULK1 pathway, as evidenced by a substantial reduction in AMPKα phosphorylation by 23.8% (*p* < 0.05), and an increase in mTOR and ULK1 phosphorylation by 49.3% (*p* < 0.05) and 25.4% (*p* < 0.01, One-way ANOVA with Dunnett’s multiple comparisons test, Fig. 10b–d), respectively. EGCG treatment successfully counteracted these effects by increasing AMPKα phosphorylation by 41.3% (*p* < 0.01) and concurrently decreasing mTOR and ULK1 phosphorylation by 39.6% (*p* < 0.05) and 16.5% (*p* < 0.01) in the hippocampus. Furthermore, SIRT1 levels, which were downregulated by 19.3% (*p* < 0.01) in the HFD group, were upregulated by 19.2% following EGCG treatment (*p* < 0.05, One-way ANOVA with Dunnett’s multiple comparisons test, Fig. 10e). These results demonstrate that EGCG can promote autophagy in the hippocampus of obese mice through the activation of the AMPK/SIRT1 and AMPK/mTOR/ULK1 signaling pathways.

## Discussion

**Fig. 11:**
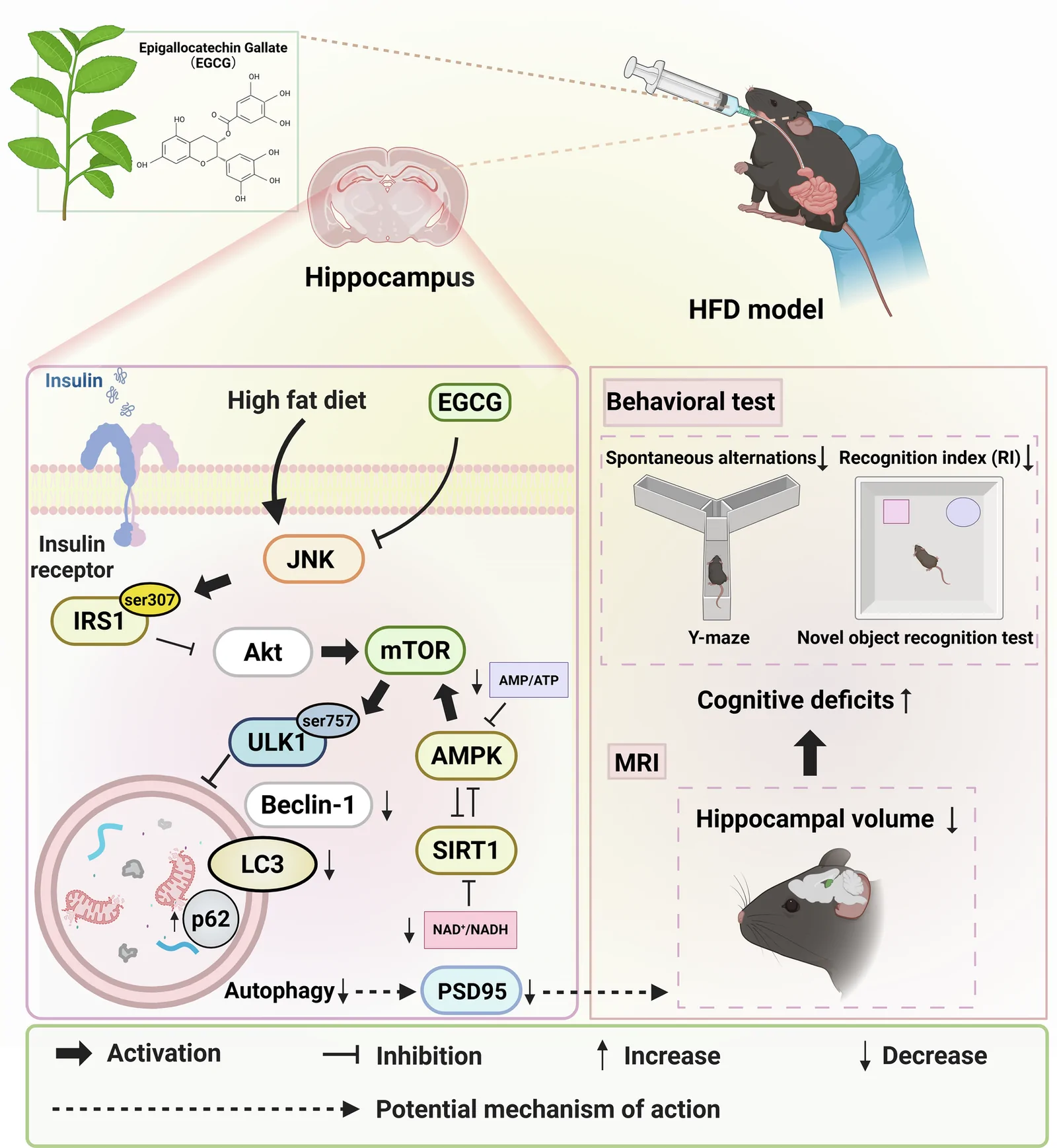
Schematic depiction of the molecular mechanisms by which EGCG alleviates hippocampal dysfunction induced by a HFD. HFD feeding induces hippocampal inflammation and insulin resistance, marked by c-Jun N-terminal kinase (JNK) activation and increased inhibitory phosphorylation of insulin receptor substrate 1 (IRS1) at Ser307, subsequently impairing downstream insulin signaling as evidenced by decreased protein kinase B (Akt) activation. Concurrently, HFD suppresses the activity of AMP-activated protein kinase (AMPK) and sirtuin 1 (SIRT1). These combined metabolic and inflammatory perturbations lead to the activation of mechanistic target of rapamycin (mTOR) and a concomitant increase in the inhibitory phosphorylation of unc-51-like autophagy activating kinase 1 (ULK1) at Serine 757. The resulting suppression of autophagy is indicated by reduced levels of microtubule-associated light chain 3 (LC3-II) and Beclin-1, alongside an accumulation of Sequestosome 1 (p62). This autophagic deficit is accompanied by the degradation of the postsynaptic density protein 95 (PSD95), contributing to hippocampal structural deterioration and the manifestation of cognitive impairment. Treatment with EGCG effectively counteracts these HFD-induced alterations by attenuating hippocampal inflammatory signaling and restoring insulin sensitivity, thereby preserving synaptic integrity and cognitive function. Upward arrows indicate increase/activation; downward arrows indicate decrease/inhibition. Created in BioRender. Ma, Z. (2026) https://BioRender.com/2cximy3. EGCG Epigallocatechin-3-Gallate, HFD High-fat diet, JNK c-Jun N-terminal kinase, IRS1 Insulin Receptor Substrate 1, Akt Protein kinase B, AMPK AMP-activated protein kinase, SIRT1 Sirtuin 1, mTOR Mechanistic target of rapamycin, ULK1 Unc-51 like autophagy activating kinase 1, LC3 microtubule-associated light chain 3, p62 Sequestosome 1, PSD95 Postsynaptic density protein 95.

In this study, we investigated whether green tea and its principal catechin, EGCG, attenuate hippocampal atrophy and cognitive impairment in obesity by integrating evidence from a population cohort and a mechanistic mouse model. In UK Biobank participants with obesity, higher green tea intake was associated with a dose-dependent, trend-level increase in hippocampal volume, showing robust stability in nonparametric bootstrap analyses. In HFD mice, a human‑achievable dose of EGCG reduced body weight and insulin resistance, preserved hippocampal volume, and improved memory performance. Mechanistically, EGCG activated the AMPK-mTOR-ULK1 and SIRT1 pathways, restored hippocampal insulin signaling, and promoted autophagy. Additionally, these changes were accompanied by attenuated inflammatory signaling (indicated by reduced JNK) and elevated expression of the synaptic protein PSD95. Together, these data support a model in which EGCG preserves neuroplasticity through the coordinated modulation of the interconnected axes of inflammation, insulin signaling, and autophagy. This integrated mechanism functionally links improvements in systemic metabolic health to the maintenance of hippocampal integrity, providing a translational rationale for dietary strategies targeting obesity‑related neurodegeneration (Fig. 11).

Our findings align with longitudinal and mechanistic evidence that hippocampal atrophy is an early imaging signature of cognitive decline and is exacerbated by obesity^7,31^. Epidemiology has associated green tea intake with lower risk of cognitive disorders and slower hippocampal atrophy^20,32^, yet effect sizes appear contingent on intake levels and cohort characteristics; studies dominated by very low consumption often report null results^33^. Consistent with this literature, our cohort analysis in obese adults suggests a dose‑responsive pattern—higher green tea intake associated with larger hippocampal volume—even though the linear trend did not reach conventional significance in our primary model. This pattern, together with the mechanistic data from mice, supports the plausibility that sufficient exposure to green tea bioactives may be required to realize neurostructural benefits.

In the animal experiments, our research strengthened causal inference by demonstrating that EGCG counters HFD‑induced hippocampal vulnerability while improving metabolic health. EGCG mitigated HFD‑induced weight gain and insulin resistance. Furthermore, MRI analysis revealed that EGCG intervention specifically restored hippocampal volume without detectable changes in cortical or whole‑brain volume, pointing to region‑specific sensitivity. This preferential hippocampal effect aligns with prior MRI reports showing that obesity and related insults disproportionately affect hippocampal subfields implicated in memory^34^. Crucially, this structural preservation translated into rescued cognitive performance in classic behavioral paradigms: In the Y‑maze and NOR tests, which are well‑validated assays for hippocampus-dependent memory performance in rodents^35,36^, EGCG significantly reversed the HFD‑induced deficits, enhancing both spontaneous alternation and Recognition Index. The concordance between volumetric rescue and behavioral improvement suggests that structural preservation reflects functionally meaningful protection. Although we performed the OFT to assess locomotor activity and anxiety-like behavior in order to exclude confounding effects on cognitive performance, additional multidimensional behavioral assessments are needed to more comprehensively rule out the influence of anxiety on cognitive function.

At the microstructural level, this macroscopic volume loss and cognitive impairment were accompanied by reduced expression of PSD95 in the hippocampus, indicating that HFD disrupts synaptic structural stability in mice. EGCG reversed these synaptic deficits^37^, providing direct structural and molecular evidence for its neuroprotective efficacy.

Mechanistically, our findings demonstrate that EGCG preserves hippocampal integrity through the coordinated modulation of inflammation, insulin signaling, and autophagy (Fig. 11). We observed that HFD significantly upregulated hippocampal phosphorylated JNK, a key inflammatory mediator known to disrupt insulin signaling via inhibitory serine phosphorylation of IRS1^11^. EGCG effectively suppressed this inflammatory axis, reversing HFD-induced Ser307 phosphorylation of IRS1 and restoring downstream Akt activation. Given that defective insulin signaling impairs autophagy induction^38^, we probed autophagy markers and upstream regulators and found that EGCG increased Beclin‑1 and the LC3‑II/LC3‑I ratio while lowering p62, consistent with enhanced autophagy. Furthermore, our results suggest that EGCG mediates this process through an AMPK-dependent program, which concurrently inhibits mTOR and augments SIRT1, suggesting a potential link between cellular energy sensing and proteostatic control^39,40^. These results support the concept of EGCG as a CRM, which can recapitulate key benefits of energy restriction including autophagy activation without requiring overt calorie reduction^25^.

Translational considerations are supported by dose contextualization across species. We selected 50 mg/kg/day EGCG based on prior efficacy in obese mice corresponding to ~4.1 mg/kg in humans by body surface area scaling^29,30^. Using group mean body weights in the cohort, the estimated human‑equivalent intakes fall within the range likely achieved by ≥2 cups/day of brewed green tea (approximately 200–300 mg EGCG per cup)^26^. Consistent with a threshold hypothesis, hippocampal volume increases were smallest in the low‑intake group and largest at or above the equivalent dose, whereas the mouse model—under tighter exposure control—showed a larger volumetric rescue. Cross‑species differences likely reflect residual confounding and exposure variability in human observers, as well as interspecies differences in absorption and metabolism; nevertheless, the shared dose‑responsiveness supports biological credibility. Notably, daily EGCG up to ~704 mg has been reported as well‑tolerated in adults, informing feasible dosing windows for future trials^41^.

Both the population cohort and the mouse model suggest that bioactive components in green tea may exert protective effects on the hippocampus. However, the nature of the exposure differs fundamentally between these approaches. In the UK Biobank, green tea intake is a complex dietary behavior. In contrast, the animal model examines EGCG as a single compound administered at a defined dose under controlled metabolic conditions. In the animal model, EGCG-treated HFD mice showed preserved hippocampal volume and memory performance. These results suggest that catechin-related signaling may mitigate obesity-associated hippocampal vulnerability by coordinately targeting inflammation, insulin signaling, and autophagy. Thus, while the population analysis establishes an association under real-world dietary conditions, the animal model provides mechanistic and causal support under controlled exposure. These findings support the biological plausibility of EGCG as a contributor to the observed population-level neuroprotective associations.

While this work has several strengths, including its integrated epidemiological analysis and preclinical validation using a human-achievable EGCG dose, several considerations help define the scope of our findings. First, our UK Biobank cohort analysis is limited by its observational design, which precludes causal inference. Second, the substantially smaller sample sizes in the tea-drinking obese subgroups (*n* = 13–100) relative to the non-tea obese group (*n* = 1732) limit the precision of the effect size estimates, although nonparametric stratified bootstrap analyses confirmed that the positive direction of the dose-response trend remained robust (see *Results* and *Methods*). Additionally, the UK Biobank dietary data capture short-term intake via 24-hour recalls but do not provide information on the duration of habitual tea consumption or its specific EGCG content, leaving open the possibility of unmeasured confounding from other tea bioactives or lifestyle factors. In our mechanistic analysis, we did not directly measure brain EGCG levels. Therefore, we could not fully disentangle the direct central actions of EGCG from the powerful secondary benefits of improved peripheral metabolism. Finally, to minimize hormonal variability^42^ and to capitalize on the greater susceptibility of male rodents to HFD-induced metabolic dysfunction^43,44^, we restricted our preclinical model to male mice. While this provided a highly controlled environment for our initial investigation, it limits the broader generalizability of our findings. Future studies with expanded sample sizes, inclusion of female subjects, and longer exposure periods are needed to investigate potential sex-specific responses to EGCG and its long-term effects. These boundaries do not diminish our current findings but rather provide a clear and rigorous roadmap for future translational research.

Future work should focus on establishing the causal role of autophagy in mediating the effects of EGCG and advancing clinical translation. First, pharmacological or genetic inhibition of autophagy in preclinical models could be employed to establish definitive causality. For example, co-administration of EGCG with autophagy inhibitors in our HFD model would directly test whether blocking autophagy abolishes EGCG’s neuroprotective and cognition-enhancing effects, thereby demonstrating that autophagy activation is a necessary mechanism for its therapeutic efficacy. Second, comprehensive behavioral test batteries are needed to rule out the possibility that the observed cognitive effects arise from anxiolytic properties of EGCG rather than direct cognitive enhancement. Finally, translational efforts could include randomized controlled trials in individuals with obesity that test standardized green tea or EGCG preparations across multiple dose tiers, with safety monitoring and pharmacokinetic profiling. The key outcomes should couple hippocampal volumetry (including subfield analyses) with sensitive cognitive endpoints, complemented by evaluation of peripheral insulin resistance. In addition, clinical studies should also examine how the treatment efficacy is modulated by interindividual factors such as diet composition, physical activity, and glycemic status.

In summary, by bridging observational neuroimaging with experimental validation, we show that obesity‑related cognitive vulnerability is associated with hippocampal volume loss and impaired insulin signaling and autophagy—pathologies ameliorated by EGCG. In obese adults, higher green tea intake is associated with larger hippocampal volume, and in obese mice, EGCG attenuates hippocampal atrophy and cognitive impairment while normalizing insulin signaling and activating autophagy pathways. These findings nominate EGCG as a mechanistically informed, readily deployable candidate for protecting hippocampal integrity in obesity, and they motivate rigorously controlled clinical trials to test cognitive and neurostructural benefits in humans.

## Methods

### UK Biobank participants

We analyzed demographic, dietary, and structural MRI data from the UK Biobank, a population cohort of over 500,000 participants recruited through UK National Health Service assessment centres between 2006 and 2010. The UK Biobank operates as a Research Tissue Bank under ethical approval from the North West–Haydock Research Ethics Committee (REC references 16/NW/0274 and 21/NW/0157). All UK Biobank participants provided written informed consent, and the study was conducted in accordance with the Declaration of Helsinki^45^. Data were accessed under an approved UK Biobank application 87507. This secondary analysis of de-identified UK Biobank data was approved by the Research Ethics Review Committee of ShanghaiTech University (ID#: E2024-038). In accordance with UK Biobank regulations, participants on official withdrawal lists were excluded. From 501,934 individuals, we identified 42,727 participants with brain MRI (acquired from 2014 onward), hippocampal volume, and whole-brain volume available. From these subjects, we excluded participants lacking BMI at the imaging visit (*n* = 1431), lacking a 24-hour dietary recall (*n* = 14,689), with documented neurological disorders (stroke, Parkinson’s disease, Alzheimer’s disease, epilepsy; *n* = 638), with cardiovascular conditions (*n* = 7118), or diabetes (*n* = 526), yielding an initial analytic sample of 18,325 participants (Fig. S1), aged 40–87 years at the imaging visit.

### Obesity classification

BMI at the imaging visit (UK Biobank field: 21001) was used for obesity classification. Per WHO criteria, normal weight was defined as 18.5–24.9 kg/m² and obesity as ≥30 kg/m²^46^.

### Exposure assessment: green tea intake

Dietary intake was collected using an interactive 24‑hour recall touchscreen questionnaire^47^. From 2009 to 2012, up to five recalls were administered. Green tea consumption (UK Biobank field: 100420) was queried as “How many cups or mugs of green tea did you drink yesterday?”. Green tea exposure was quantified on a cup-based scale, which represents the highest resolution available in the UK Biobank. In the present study, “6+” was coded as 7 for analysis. For each participant, the mean daily intake was computed as total cups across valid recalls divided by the number of recalls (UK Biobank field: 20078). This measure was then categorized into distinct groups for subsequent analysis.

### Group definitions

From the initial analytic sample (*n* = 18,325), participants were excluded due to missing covariate data (*n* = 1666) or according to BMI and green tea intake categories (*n* = 7500), including underweight (BMI < 18.5 kg/m²; *n* = 168), overweight (25 ≤ BMI < 30 kg/m²; *n* = 6388), and normal-weight green tea consumers (*n* = 944). The remaining participants were grouped by BMI and green tea intake as follows: (1) Normal‑weight and no tea (NW‑NT; *n* = 7298), (2) Obese and no tea (OB‑NT; *n* = 1732), (3) Obese and low intake (OB‑LT; >0 to ≤1 cup/day; *n* = 100), (4) Obese and moderate intake (OB‑MT; >1 to ≤2 cups/day; *n* = 13), (5) Obese and high intake (OB‑HT; >2 cups/day; *n* = 16).

### MRI acquisition and imaging phenotype extraction

All scans were acquired on identical Siemens Skyra 3.0 T systems (VD13A SP4) with a standard 32‑channel head coil at three imaging centers. T1‑weighted structural images were collected using a 3D MPRAGE sequence with the following parameters: repetition time (TR) = 2000 ms; inversion time = 880 ms; matrix size = 208 × 256 × 256; voxel size = 1 × 1 × 1 mm³; iPAT=2^48^. Raw T1w MPRAGE images were preprocessed using the UK Biobank’s structural pipeline^49^, and hippocampal volume was obtained using FMRIB’s Integrated Registration and Segmentation Tool (FIRST)^50^. For this study, total hippocampal volume was calculated as the sum of the left (UK Biobank field: 25019) and right (UK Biobank field: 25020) volumes.

### Statistics: UKB green tea and hippocampal volume

Our epidemiological analysis employed two complementary statistical models. Model 1 evaluated the fundamental impact of obesity on hippocampal volume across the full cohort. Model 2 assessed the dose-response relationship between green tea intake and hippocampal volume exclusively within the obese subgroups. Model 2 was supplemented by nonparametric sensitivity analyses.

Both models were adjusted for relevant demographic and lifestyle covariates that may influence hippocampal volume: age at the time of the first imaging visit (UK Biobank field: 21003), sex (UK Biobank field: 31), head size scaling factor (UK Biobank field: 25000)^51^, and educational background (UK Biobank field: 6138). Lifestyle-related variables, including average time spent on moderate physical activity (UK Biobank field: 894), smoking status (UK Biobank field: 20116), and alcohol status (UK Biobank field: 20117), were also included. “Prefer not to answer” responses were coded as missing data. To adjust for potential batch effects associated with MRI acquisition, scanner site (UK Biobank field: 54) was included as a categorical covariate.

Continuous covariates (age, head size scaling factor, and duration of moderate activity) were centered at their sample means to aid interpretability of the model intercept. Categorical factors were modeled with contrast coding; the reference levels were set a priori (e.g., “NW-NT” for group; “female” for sex; the most frequent site, “Cheadle”, for scanner site; “None” for education background; “Never” for smoking status; “Never” for alcohol status). Missingness was handled by listwise deletion.

Before model fitting, numeric covariates were screened for pairwise correlation (|r | > 0.50) to detect multicollinearity. Here, standing height (UK Biobank field: 50) was not included due to high correlation with the head size scaling factor (r = 0.60); no other prespecified covariates exceeded this threshold and all were retained. We also screened for potential interactions between covariates using nested ordinary least squares (OLS) models and ANOVA F-tests. No significant interaction terms met the α = 0.05 threshold (including Sex × Group, *p* = 0.63); therefore, no interactions were included in the final models.

Model 1: Full cohort analysis. The relationship between group and hippocampal volume was formulated in Wilkinson notation as:

1 $$\begin{array}{l}Hippocampal\,volume\,=\,{{\rm{\beta }}}_{0}\,+\,{{\rm{\beta }}}_{1}\cdot \,Group\\ +\,{{\rm{\beta }}}_{2}\cdot \mathrm{Age}\,\mathrm{at}\,\mathrm{imaging}\,\mathrm{visit}+{{\rm{\beta }}}_{3}\cdot \mathrm{Duration}\,\mathrm{of}\,\mathrm{moderate}\,\mathrm{activity}\\ +\,{{\rm{\beta }}}_{4}\cdot \mathrm{Head}\,\mathrm{size}\,\mathrm{scaling}\,\mathrm{factor}+{{\rm{\beta }}}_{5}\cdot \mathrm{Sex}+{{\rm{\beta }}}_{6}\cdot \mathrm{Educational}\,\mathrm{qualification}\\ +\,{{\rm{\beta }}}_{7}\cdot \mathrm{Scanner}\,\mathrm{site}+{{\rm{\beta }}}_{8}\cdot \mathrm{Smoking}\,\mathrm{status}+{{\rm{\beta }}}_{9}\cdot \mathrm{Alcohol}\,\mathrm{status}+{\rm{\varepsilon }}\end{array}$$

A robust linear model (RLM)^52,53^, with Huber weighting, using the R package MASS (v7.3-65), was employed as the primary analysis method to mitigate the influence of outliers and relax the normality assumption of residuals. An ANCOVA omnibus test for the group effect was conducted using a robust R² heuristic. After fitting the RLM, we obtained EMMs for each group with the R package emmeans (v1.11.2). Unlike raw descriptive averages, which are inherently biased by unequal sample sizes, EMMs mathematically evaluate predictions over a balanced reference grid, adjusting for both group size imbalance and covariate distributions^54^. EMMs were compared with Tukey’s multiplicity adjustment for all pairwise group contrasts. Standard errors for all EMM contrasts were derived via matrix multiplication of the specified contrast weights and the model’s variance-covariance matrix, and *p*-values were determined using an asymptotic z-test.

Model 2: Dose-response analysis within the obese cohort. To assess a potential dose–response of tea intake among individuals with obesity, we restricted the sample to the four obese categories (OB-NT, OB-LT, OB-MT, OB-HT). For this analysis, continuous BMI (centered) and average daily coffee consumption (centered) were added as covariates. BMI was included to statistically control for the observed BMI differences between obese groups (Table S1), and coffee consumption was included to control for its established independent associations with hippocampal volume and neuroprotective outcomes^55^. Coffee consumption was defined as the mean daily number of cups, calculated by summing all reported types of coffee (UK Biobank fields: 100250, 100270, 100290, 100300, 100310, and 100330) across valid dietary recalls and dividing by the number of completed recalls (UK Biobank Field 20078).

These additional covariates were also screened for multicollinearity (|r | > 0.50; no exclusions required) and for interactions with tea dose (including Sex × Group, BMI × Group, and Coffee × Group; all *p* > 0.05). The dose-response model was therefore formulated as:

2 $$\begin{array}{l}Hippocampal\,volume\,=\,{{\rm{\beta }}}_{0}\,+\,{{\rm{\beta }}}_{1}\cdot \,Group\\ +\,{{\rm{\beta }}}_{2}\cdot \mathrm{Age}\,\mathrm{at}\,\mathrm{imaging}\,\mathrm{visit}+{{\rm{\beta }}}_{3}\cdot \mathrm{Duration}\,\mathrm{of}\,\mathrm{moderate}\,\mathrm{activity}\\ +\,{{\rm{\beta }}}_{4}\cdot \mathrm{Head}\,\mathrm{size}\,\mathrm{scaling}\,\mathrm{factor}+{{\rm{\beta }}}_{5}\cdot \mathrm{Sex}+{{\rm{\beta }}}_{6}\cdot \mathrm{Educational}\,\mathrm{qualification}\\ +\,{{\rm{\beta }}}_{7}\cdot \mathrm{Scanner}\,\mathrm{site}+{{\rm{\beta }}}_{8}\cdot \mathrm{Smoking}\,\mathrm{status}+{{\rm{\beta }}}_{9}\cdot \mathrm{Alcohol}\,\mathrm{status}+\,{{\rm{\beta }}}_{10}\cdot \mathrm{BMI}+{{\rm{\beta }}}_{11}\cdot \mathrm{Coffee}\,\mathrm{cups}+{\rm{\varepsilon }}\end{array}$$

Tea dose was modeled as an ordered factor (no, low, moderate, high) in an RLM with these updated covariates. To evaluate the dose-response relationship, the adjusted EMMs were analyzed using polynomial contrasts to test for a linear trend. The standard error for this linear contrast was mathematically derived from the model’s variance-covariance matrix, and the *p*-value for the linear trend was calculated using an asymptotic z-test. All pairwise comparisons across the dose groups were Tukey-adjusted.

To evaluate the stability of the linear dose-response trend given the unequal group sizes, we performed two nonparametric stratified bootstrap analyses, each with 2000 resamples. First, resampling was strictly stratified within the original obesity-tea categories to preserve exact group proportions across iterations. From the resulting bootstrap distribution of the linear trend, we extracted the proportion of iterations yielding a positive linear coefficient. Second, to maximize statistical power and stabilize variance, we collapsed all tea consumers into a single group (OB-Tea, *n* = 129) and compared them against non-consumers (OB-NT, *n* = 1732) using the same stratified bootstrap approach, extracting the proportion of iterations yielding a positive volume difference.

### Animal models and study design

All procedures were approved by the Animal Research Center of Shanghai University of Traditional Chinese Medicine (PZSHUTCM2406260001). Male C57BL/6 mice (6 weeks, 18–25 g) were housed under standard conditions (12 h light/dark; 25 °C) with ad libitum access to food and water.

To validate the findings observed in the human cohort and further explore the underlying molecular mechanisms, we established a mouse model simulating HFD-induced obesity and EGCG intervention. After one week of acclimation, a total of 24 mice were randomly assigned to treatment groups using simple randomization. An online random number generator (GIGAcalculator) was used to select 8 identifiers^56^. These mice were allocated to the Control group (standard chow diet), while the remaining 16 mice constituted the HFD group (60 kcal% fat). At the end of week 8, the 16 HFD-fed mice were recoded (1-16). A second round of simple randomization was performed using the same online generator to select 8 mice to continue as the HFD group, while the remaining 8 mice were assigned to the HFD + EGCG group. From weeks 8 to 12, the HFD + EGCG group received EGCG (50 mg/kg body weight/day, dissolved in saline) via daily oral gavage, while the Control and HFD groups received an equal volume of saline vehicle. A parallel cohort (Control *n* = 9; HFD *n* = 7; HFD + EGCG *n* = 8) underwent identical modeling for MRI. Diets were sourced from TROPHIC (standard: 10 kcal% fat, TP2330055ACX; HFD: 60 kcal% fat, TP2330055AX). EGCG (HPLC ≥ 98%) was sourced from Sichuan Weikeqi Biotechnology Co., Ltd., China.

### Metabolic phenotyping

Intraperitoneal glucose tolerance tests (IGTTs) were conducted between weeks 8 and 12 after overnight fasting (water *ad libitum*). A 10% glucose solution (1 g/kg; Leagene R00601) was injected intraperitoneally, and blood glucose was measured at 0, 30, 60, 90, and 120 min^57^. Area under the curve (AUC) was computed from the time–glucose profile.

For the homeostasis model assessment of insulin resistance (HOMA‑IR), fasting blood glucose (FBG) was measured by glucometer and fasting insulin (FIN) by ELISA (Nanjing Jiancheng Bioengineering Research Institute Co., Ltd., China). HOMA-IR was determined using the following equation^58^:

3 $$\mathrm{HOMA-IR}=\frac{\mathrm{FBG}\,(\mathrm{mmol/L})\times \mathrm{FIN}\,(\mu\mathrm{IU/mL})}{22.5}$$

### Mouse brain structural MRI acquisition and segmentation

Anesthesia was induced with 3.5% isoflurane and maintained at 1.5% during initial preparation. For MRI scanning, dexmedetomidine (0.025 mg/kg, i.p.) was administered, and isoflurane was reduced to 0.3–0.5% to maintain a stable respiratory rate of 140–150 breaths/min, which was monitored throughout the MRI acquisition using a ventral pressure sensor (SA Instruments, Stony Brook, NY, USA).

High-resolution T2‑weighted images with whole-brain coverage were acquired on a 9.4 T Bruker BioSpec 94/30 system equipped with a four‑channel cryogenic surface coil (CryoProbe). A Turbo‑RARE sequence was used with the following parameters: TR = 3600 ms; effective echo time = 35 ms; RARE factor = 8; matrix size = 250 × 200; number of slices = 40; voxel size = 0.08 × 0.08 × 0.40 mm³; excitation flip angle = 90°; refocusing flip angle = 180°.

Manual segmentation of the hippocampus, cerebral cortex, and whole brain was performed in ITK‑SNAP. Segmentation was guided by the Allen Mouse Brain Common Coordinate Framework (v3) atlas^59^. The process was conducted slice-by-slice on 40 contiguous coronal slices from high-resolution structural MRI images that provided full coverage of the mouse brain’s anterior-posterior extent. The volume (mm³) of each resulting region of interest (ROI) was then calculated. Relative hippocampal volume was calculated as hippocampal volume divided by whole-brain volume. Relative cortical volume was calculated as cerebral cortex volume divided by whole-brain volume. Figure 5b provides a three-dimensional schematic of the hippocampus and cortex in the mouse brain for anatomical reference.

### Behavioral testing

To assess short-term spatial working memory, mice were tested in a Y-maze. At the beginning of the test, one arm of the maze was blocked, and the mouse was placed in the center for a 10-minute free exploration period. One hour later, the blockage was removed, allowing the mouse to freely explore all three arms for another 5 minutes^60^. The sequence of arm entries was recorded. A “spontaneous alternation” was defined as a sequence of entries into three different arms on consecutive choices. The percentage of spontaneous alternation was calculated to evaluate memory performance using the following formula^61^:

4 $${Spontaneous}\,{alternation}\,{rate}\,( \% )=\frac{{Number}\,{of}\,{alternations}}{{Total}\,{number}\,{of}\,{arm}\,{entries}-2}\times 100$$

Hippocampal-dependent recognition memory of mice was evaluated through the NOR test, which consists of three phases^62^: (1) Habituation: Mice were first habituated to the empty testing arena by allowing them to explore it freely for 5 min (2) Familiarization: The next day, mice were placed back into the arena, which now contained two identical objects, and were allowed to explore for 10 minutes. (3) Testing: On the final day, one of the identical objects was replaced with a novel object. Mice were returned to the arena, and their exploration behavior was recorded for 10 minutes. A Recognition Index was calculated to quantify memory, reflecting the mouse’s preference for the novel object over the familiar one^63^:

5 $${RI}\,( \% )=\frac{{Time}\,{exploring}\,{novel}\,{object}}{{Time}\,{exploring}\,{novel}\,{object}\,+\,{Time}\,{exploring}\,{familiar}\,{object}}\times 100$$

To assess general locomotor activity and anxiety-like behavior, the OFT was used. The mice were placed in a non-transparent polypropylene arena (50 × 50 × 40 cm) and allowed to explore freely for 5 minutes. The total distance traveled (cm) and the time spent in center (30 × 30 cm) were analyzed to reflect the mice’s anxiety levels^64,65^.

### Serum and tissue collection

After the final behavioral test, mice were fasted for 12 h. Fasting blood glucose was measured from the tail vein. The animals were anesthetized with sodium pentobarbital (40 mg/kg, i.p.). Blood was collected from the carotid artery, after which the mice were euthanized. Serum was separated by centrifugation at 3000 × g for 15 min at 4 °C and stored at −20 °C for subsequent analysis. The hippocampus was rapidly dissected on an ice-cold surface, snap-frozen in liquid nitrogen, and stored at −80 °C for further study.

### Transmission electron microscopy

For electron microscopic analysis, mice were deeply anesthetized with sodium pentobarbital (i.p.) and transcardially perfused with ice-cold saline, followed by electron microscopy-grade fixative (G1102, Wuhan Servicebio Technology Co., Ltd.). Following brain extraction, the hippocampal CA3 region was dissected into 1 × 2 × 3 mm blocks. These blocks were additionally fixed in fresh electron microscopy-grade fixative at 4 °C for 2–4 h and then transported on ice under light protection to Servicebio for processing. Subsequent steps were performed according to an established protocol^66^. Briefly, samples were fixed in 0.1 M phosphate buffer (PB, pH 7.4), rinsed, dehydrated through a graded ethanol series, and embedded in epoxy resin. Ultrathin sections (60–80 nm) were prepared, stained with uranyl acetate and lead citrate, and examined using a Tecnai G2 20 TWIN transmission electron microscope (FEI, USA).

### Immunohistochemistry

Immunohistochemistry on hippocampal sections was performed as previously described^67^. Mice were perfused-fixed with 4% paraformaldehyde (PFA) under deep anesthesia. Following extraction, brains were post-fixed overnight in 4% PFA at 4 °C, and subsequently cryoprotected in 10% and 30% sucrose solutions and embedded in optimal cutting temperature (OCT) compound (Yeasen Biotech, Shanghai). Coronal sections (20μm) were cut on a cryostat. Sections were blocked with 5% bovine serum albumin for 30 minutes, then incubated overnight at 4 °C with primary antibody against NeuN (1:300; Abcam, ab177487). After washes, sections were incubated with fluorescent secondary antibodies, counterstained with DAPI and imaged using an Olympus VS120 (Tokyo, Japan) fluorescence microscope. The density of NeuN^+^ cells in the hippocampal CA3 region was quantified using ImageJ (v1.53k) software.

### Western blotting

A subset of 4-5 samples was randomly selected from the total cohort of eight for the Western blot. Hippocampi were lysed in RIPA buffer containing protease/phosphatase inhibitors. Proteins were separated by SDS‑PAGE (4–20% FuturePAGE™ gels; ACE, Changzhou) and transferred to PVDF membranes. Membranes were blocked in 5% nonfat milk or rapid blocking solution for 1 h, incubated with primary antibodies overnight at 4 °C, followed by horseradish peroxidase‑conjugated secondary antibodies. The blots were displayed using a Super ECL Detection Reagent and visualized using a chemiluminescence imaging system (GE Healthcare, Chicago, IL, USA). Quantitative densitometry of protein bands was performed using ImageJ (v1.53k) software. Protein signals were normalized to the β-actin signal.

### Antibodies and reagents for Western blotting

Primary antibodies: p‑IRS1 (Ser307) (3 μg/mL; Abcam ab5599), IRS1 (1:250; Invitrogen PA1‑1057), p‑Akt (Ser473) (1:2000; CST #4060S), Akt (1:1000; CST #9272S), p-JNK (Thr183/Tyr185) (1:1000, CST #4668S), JNK (1:1000, CST #9252S), p‑mTOR (Ser2448) (1:1000; Abcam ab109268), mTOR (1:1000; Abcam ab134903), p‑AMPKα (Thr183/Thr172) (1:1000; Abcam ab133448), AMPKα (1:1000; Abcam ab271188), SIRT1 (1:1000; Abcam ab110304), p‑ULK1 (Ser757) (1:1000; Abcam ab229909), ULK1 (2 μg/mL; Abcam ab167139), SQSTM1/p62 (1:10,000; Abcam ab109012), Beclin‑1 (1:2000; Abcam ab207612), LC3B (LC3‑II/LC3‑I) (1:2000; Abcam ab192890), PSD95 (1:1000; Abcam ab13552/ab99009), β‑Actin (1:5000; Bioss bs‑0061 R).

Secondary antibodies: Goat anti‑Mouse IgG (1:1000; Jackson 115‑035‑003) and Goat anti‑Rabbit IgG (1:1000; Jackson 111‑035‑003).

Additional reagents: BCA protein assay kit, rapid blocking and ECL developing reagents (Yeasen Biotech, Shanghai); Insulin ELISA kit (Nanjing Jiancheng Bioengineering).

### Statistical analysis for mouse experiments

Data were analyzed using GraphPad Prism (v.9.0) and are presented as mean ± SEM. The Shapiro–Wilk test was used to assess normality. An unpaired t-test was used to compare the initial body weight between groups prior to the experiment. For comparisons at the 8-week time point before EGCG intervention, Welch’s t-test was applied to body weight (due to unequal variances, confirmed by F-test), while the Mann–Whitney U test was used for the IGTT AUC, as well as for body weight and AUC at the initial grouping of the HFD and HFD + EGCG groups, due to non-normality. Weight and AUC in the intervention randomization groups were also analyzed using the Mann–Whitney U test due to non-normality. Following EGCG intervention, multiple-group comparisons were performed as follows: one-way ANOVA with Dunnett’s test was applied to data conforming to parametric assumptions (body weight, IGTT AUC, fasting blood glucose in HOMA-IR, NOR acquisition, the total distance traveled in OFT, relative cortical volume, hippocampal protein expression, and NeuN^+^ cell density); one-way ANOVA with Bonferroni’s test was applied to relative hippocampal volume; the Kruskal–Wallis test with Dunn’s test was used for non-parametric data (HOMA-IR, fasting insulin in HOMA-IR, Y-maze spontaneous alternation, time in center during OFT, and whole-brain volume); and Brown–Forsythe and Welch ANOVA with Dunnett’s T3 test were employed for NOR test results, which violated homogeneity of variance.

## Supplementary information

Supplementary Information

## Data Availability

The UK Biobank datasets analyzed in the current study are not publicly available due to UK Biobank regulations, but they can be requested via the Access Management System at https://ams.ukbiobank.ac.uk/ams/. All other data supporting the findings of this study are included within the article and its Supplementary Information file. Additional mouse MRI datasets analyzed during the current study are available from the corresponding authors upon reasonable request.
