# Supplementary material for: Green tea catechin EGCG attenuates hippocampal atrophy and cognitive impairment in obesity via autophagy signaling
Source: NPJ Sci Food. 2026 Jun 4;10:274. doi: 10.1038/s41538-026-00914-4 (PMC13554131; doi:10.1038/s41538-026-00914-4)
Supplement: Supplementary file 1 [file 41538_2026_914_MOESM1_ESM.docx]

**Supplementary Information for**

**Green tea catechin EGCG attenuates hippocampal atrophy and cognitive impairment in obesity via autophagy signaling**

Kunyi Huang^1,2,3^**^†^**, Xin Li^1,4^**^†^**, Yujie Chen^2^**^†^**, Yiyun Qi^1^, Weiying Zhang^2^, Jia Ee Tan^2^, Jia Ying Chan^2^, Jiashuo Lu^2^, Fangyi Hao^1,4^, Jiayi Shi^1,4^, Shuting Yan^1^, Zhenzhen Huang^5^, Xiaojun Wu^3^*****, Qi Xu^2^*****, Zhiwei Ma^1,6,7^*****

**Affiliations**

1. School of Biomedical Engineering, ShanghaiTech University, Shanghai 201210, China

2. School of Public Health, Shanghai University of Traditional Chinese Medicine, Shanghai 201203, China

3. State Key Laboratory of Discovery and Utilization of Functional Components in Traditional Chinese Medicine, Shanghai Key Laboratory of Compound Chinese Medicines, Institute of Chinese Materia Medica, Shanghai University of Traditional Chinese Medicine, Shanghai 201203, China

4. School of Life Science and Technology, ShanghaiTech University, Shanghai 201210, China

5. Laboratory Animal Center, Shanghai University of Traditional Chinese Medicine, Shanghai 201203, China

6. State Key Laboratory of Advanced Medical Materials and Devices, ShanghaiTech University, Shanghai 201210, China

7. Shanghai Clinical Research and Trial Center, Shanghai 201210, China

**†**: Kunyi Huang, Xin Li, and Yujie Chen contributed equally to this work.

*Corresponding authors:

Xiaojun Wu (xiaojunwu320@126.com)

Qi Xu (isuxuqi@163.com)

Zhiwei Ma (zma.qualia@gmail.com)

This file includes:

Table S1 to S5: page 2 – 6

Figure S1 to S5: page 7 – 12

Western blot raw data: 13 – 40**Table S1.** Demographic and health characteristics of UK Biobank groups.

| Group | Sample size | Sex | | Age, mean ± SD | BMI, mean ± SD |
| --- | --- | --- | --- | --- | --- |
|  |  | Male | Female |  |  |
| NW-NT | 7298 | 2639 | 4659 | 62.978 ± 7.622 | 22.679 ± 1.565 |
| OB-NT | 1732 | 712 | 1020 | 61.465 ± 7.609 | 32.987 ± 3.087 |
| OB-LT | 100 | 28 | 72 | 61.780 ± 6.885 | 32.593 ± 2.310 |
| OB-MT | 13 | 6 | 7 | 60.154 ± 7.820 | 32.347 ± 1.659 |
| OB-HT | 16 | 5 | 11 | 59.250 ± 6.309 | 35.167 ± 5.904 |

Abbreviations:

SD: Standard deviation;

BMI: Body mass index;

NW-NT: Normal weight with no tea intake;

OB-NT: Obese with no tea intake;

OB-LT: Obese with low green tea intake;

OB-MT: Obese with moderate green tea intake;

OB-HT: Obese with high green tea intake.

**Table S2.** Adjusted pairwise group comparisons based on a robust linear regression model.

| Group | Coefficient | SE | Z-value | P-value |
| --- | --- | --- | --- | --- |
| NW-NT vs. OB-NT | 55.80 | 19.60 | 2.85 | 0.04 |
| NW-NT vs. OB-MT | -151.10 | 200.00 | -0.75 | 0.94 |
| NW-NT vs. OB-LT | -21.20 | 72.70 | -0.29 | 1.00 |
| NW-NT vs. OB-HT | -251.90 | 181.00 | -1.40 | 0.63 |
| OB-NT vs. OB-MT | -206.80 | 201.00 | -1.03 | 0.84 |
| OB-NT vs. OB-LT | -77.00 | 74.20 | -1.04 | 0.84 |
| OB-NT vs. OB-HT | -307.70 | 181.00 | -1.70 | 0.44 |
| OB-MT vs. OB-LT | 129.80 | 213.00 | 0.61 | 0.97 |
| OB-MT vs. OB-HT | -100.80 | 270.00 | -0.37 | 1.00 |
| OB-LT vs. OB-HT | -230.60 | 194.00 | -1.19 | 0.76 |

Post-hoc pairwise comparisons were conducted using robust linear regression (Eq. 1 in the main text), with Tukey's method applied to adjust p-values for multiple testing. The model was adjusted for the following covariates: age at imaging visit, sex, head size scaling factor, moderate physical activity duration, scanner site, smoking status, and alcohol consumption status.

Abbreviations:

SE: Standard error;

NW-NT: Normal weight with no tea intake;

OB-NT: Obese with no tea intake;

OB-LT: Obese with low green tea intake;

OB-MT: Obese with moderate green tea intake;

OB-HT: Obese with high green tea intake.

**Table S3.** Adjusted hippocampal volume by tea intake level in obese individuals.

| Group | Adjusted Mean Volume (mm³) | SE | 95% CI |
| --- | --- | --- | --- |
| OB-NT | 7650 | 119 | [7416, 7884] |
| OB-LT | 7719 | 136 | [7452, 7986] |
| OB-MT | 7849 | 238 | [7383, 8314] |
| OB-HT | 7976 | 222 | [7540, 8411] |

Values were estimated marginal means (95% CI) using robust linear regression (Eq. 2 in the main text) adjusted for age at imaging visit, sex, BMI, coffee consumption, head size scaling factor, moderate physical activity duration, scanner site, smoking status, and alcohol consumption status.

Abbreviations:

SE: Standard error;

CI: Confidence interval;

BMI: Body mass index;

OB-NT: Obese with no tea intake;

OB-LT: Obese with low green tea intake;

OB-MT: Obese with moderate green tea intake;

OB-HT: Obese with high green tea intake.

**Table S4.** One-sample t-test results for Recognition Index (RI) during the NOR acquisition phase compared to the 50% threshold.

| Group | Recognition Index (RI) (%, Mean ± SEM) | t-value | p-value |
| --- | --- | --- | --- |
| Control | 49.07 ± 2.68 | 0.35 | 0.74 |
| HFD | 46.12 ± 4.13 | 0.94 | 0.38 |
| HFD+EGCG | 49.01 ± 2.05 | 0.48 | 0.64 |

Abbreviations:

EGCG: Epigallocatechin-3-gallate;
HFD: High-fat diet;

HFD+EGCG: High-fat diet combined with epigallocatechin-3-gallate;

SEM: Standard error of the mean.

**Table S5.** One-sample t-test results for Recognition Index (RI) during the NOR testing phase compared to the 50% threshold.

| Group | Recognition Index (RI) (%, Mean ± SEM) | t-value | p-value |
| --- | --- | --- | --- |
| Control | 62.52 ± 4.02 | 3.12 | 0.02 |
| HFD | 50.10 ± 1.12 | 0.09 | 0.93 |
| HFD+EGCG | 65.09 ± 4.62 | 3.26 | 0.01 |

Abbreviations:

EGCG: Epigallocatechin-3-gallate;
HFD: High-fat diet;

HFD+EGCG: High-fat diet combined with epigallocatechin-3-gallate;

SEM: Standard error of the mean.

**Figure S1.** **Participant selection and group definition based on BMI and green tea intake in the UKB cohort.**

Abbreviations:

UKB: UK Biobank

BMI: Body mass index;

NW-NT: Normal weight and no tea intake;

OB-NT: Obese and no tea intake;

OB-LT: Obese and low green tea intake;

OB-MT: Obese and moderate green tea intake;

OB-HT: Obese and high green tea intake.

**Figure S2. Baseline body weight, glucose AUC, and changes in body weight over time. (a)** Initial body weight (Unpaired t-test; Control: n = 8; HFD: n = 16). **(b)** Weight changes during the modeling period (0–8 weeks; Control: n = 8; HFD: n = 16). **(c)** Body weight at week 8, immediately before EGCG intervention/randomization (Mann–Whitney U test; n = 8/group). **(d)** Glucose AUC derived from the IGTT at week 8 (Mann–Whitney U test; n = 8/group; each dot represents one mouse’s AUC value). **(e)** Weight changes during the intervention period (8–12 weeks; n = 8/group). Data are mean ± SEM. Comparisons are versus the HFD group.

Abbreviations:

EGCG: Epigallocatechin-3-gallate;
HFD: High-fat diet;

HFD+EGCG: High-fat diet combined with epigallocatechin-3-gallate;

IGTT: Intraperitoneal glucose tolerance test;

AUC: Area under the curve;

SEM: Standard error of the mean.

**Figure S3.** **Open field test (OFT) shows no significant differences in locomotor activity or anxiety-like behavior among groups.** **(a)** Total distance traveled in OFT (One‑way ANOVA with Dunnett’s multiple comparisons test; n = 8/group). **(b)** Time spent in center of OFT (Kruskal–Wallis test with Dunn’s multiple comparisons test; n = 8/group). Data are mean ± SEM. Comparisons are versus the HFD group.

Abbreviations:
EGCG: Epigallocatechin-3-gallate;

HFD: High-fat diet;

HFD+EGCG: High-fat diet combined with epigallocatechin-3-gallate;

OFT: Open field test;

SEM: Standard error of the mean.

**Figure S4. EGCG protects against HFD-induced loss of NeuN^+^ neurons in the hippocampal CA3 region. (a)** Representative immunofluorescence images of NeuN expression in CA3. Coronal brain sections were immunostained for NeuN (red) to visualize mature neurons, and nuclei were counterstained with DAPI (blue). Scale bar: 100 µm. **(b)** Quantification of NeuN^+^ cell density in hippocampal CA3 (NeuN^+^ cells per mm²; one-way ANOVA with Dunnett’s multiple comparisons test; n = 3 mice/group; each dot represents one mouse). Data are mean ± SEM. Comparisons are versus the HFD group.

Abbreviations:

EGCG: Epigallocatechin-3-gallate;

HFD: High-fat diet;

HFD+EGCG: High-fat diet combined with epigallocatechin-3-gallate;

SEM: Standard error of the mean.

**Figure S5.** **Ultrastructural characterization of hippocampal autophagy.** Representative transmission electron microscopy (TEM) images of the CA3 hippocampal region from the indicated experimental groups, showing autophagic structures. Yellow arrows point to autophagic vacuoles, including double-membrane autophagosomes and autolysosomes. Scale bars: 5 µm (first row) and 2 µm (second row).

Abbreviations:

EGCG: Epigallocatechin-3-gallate;

HFD: High-fat diet;

HFD+EGCG: High-fat diet combined with epigallocatechin-3-gallate.

Western blot raw data

| Panels from main figures | Western blot raw data |
| --- | --- |
| Fig 7a PSD95/β-actin   | Raw data of Fig 7a PSD95/β-actin  180kDa  130kDa  100kDa  72kDa  55kDa  43kDa  33kDa  25kDa  17kDa  180kDa  130kDa  100kDa  72kDa  55kDa  43kDa  33kDa  25kDa  17kDa  Control  HFD  HFD+EGCG  Control  HFD  HFD+EGCG  43kDa  33kDa  β-actin  PSD95  100kDa  72kDa  180kDa  130kDa  100kDa  72kDa  55kDa  43kDa  33kDa  25kDa  17kDa  180kDa  130kDa  100kDa  72kDa  55kDa  43kDa  33kDa  25kDa  17kDa  Control  HFD  HFD+EGCG  Control  HFD  HFD+EGCG  43kDa  33kDa  β-actin  PSD95  100kDa  72kDa  **Repeat 1**  **Repeat 2**  PSD95  β-actin  180kDa  130kDa  100kDa  72kDa  55kDa  43kDa  33kDa  25kDa  17kDa  180kDa  130kDa  100kDa  72kDa  55kDa  43kDa  33kDa  25kDa  17kDa  Control  HFD  HFD+EGCG  Control  HFD  HFD+EGCG  43kDa  33kDa  β-actin  PSD95  100kDa  72kDa  **Repeat 3**  **Note: Due to excessive exposure of the final PSD95 dark-field image, resulting in poor actin visualization, the entire membrane was additionally displayed with its developed image on a white background.**  PSD95  β-actin  180kDa  130kDa  100kDa  72kDa  55kDa  43kDa  33kDa  25kDa  17kDa  180kDa  130kDa  100kDa  72kDa  55kDa  43kDa  33kDa  25kDa  17kDa  Control  HFD  HFD+EGCG  Control  HFD  HFD+EGCG  43kDa  33kDa  β-actin  PSD95  100kDa  72kDa  **Repeat 4**  **Note: Due to excessive exposure of the final PSD95 dark-field image, resulting in poor actin visualization, the entire membrane was additionally displayed with its developed image on a white background.** |
| Fig 8a p-JNK/JNK  180kDa  130kDa  100kDa  72kDa  55kDa  43kDa  33kDa  25kDa  180kDa  130kDa  100kDa  72kDa  55kDa  43kDa  33kDa  25kDa  p-JNK  Control  HFD  HFD+EGCG  Control  HFD  HFD+EGCG  Control  HFD  HFD+EGCG  Control  HFD  HFD+EGCG  43kDa  55kDa  180kDa  130kDa  100kDa  72kDa  55kDa  43kDa  33kDa  25kDa  JNK  55kDa  33kDa  43kDa  β-actin  55kDa  33kDa  43kDa  180kDa  130kDa  100kDa  72kDa  55kDa  43kDa  33kDa  25kDa | Raw data of Fig 8a p-JNK/JNK  **Repeat 2**  **Repeat 1**  33kDa  130kDa  100kDa  72kDa  55kDa  43kDa  33kDa  25kDa  17kDa  8kDa  β-actin  55kDa  33kDa  43kDa  130kDa  100kDa  72kDa  55kDa  43kDa  33kDa  25kDa  17kDa  JNK  55kDa  33kDa  43kDa  130kDa  100kDa  72kDa  55kDa  43kDa  33kDa  25kDa  17kDa  130kDa  100kDa  72kDa  55kDa  43kDa  33kDa  25kDa  17kDa  p-JNK  Control  HFD  HFD+EGCG  Control  HFD  HFD+EGCG  55kDa  33kDa  43kDa  **Repeat 3**  180kDa  130kDa  100kDa  72kDa  55kDa  43kDa  33kDa  25kDa  17kDa  180kDa  130kDa  100kDa  72kDa  55kDa  43kDa  33kDa  25kDa  17kDa  β-actin  55kDa  33kDa  43kDa  p-JNK  Control  HFD  HFD+EGCG  Control  HFD  HFD+EGCG  43kDa  55kDa  33kDa  180kDa  130kDa  100kDa  72kDa  55kDa  43kDa  33kDa  25kDa  17kDa  JNK  55kDa  33kDa  43kDa  180kDa  130kDa  100kDa  72kDa  55kDa  43kDa  33kDa  25kDa  17kDa  **Repeat 4**  180kDa  130kDa  100kDa  72kDa  55kDa  43kDa  33kDa  25kDa  17kDa  JNK  55kDa  33kDa  43kDa  180kDa  130kDa  100kDa  72kDa  55kDa  43kDa  33kDa  25kDa  17kDa  p-JNK  Control  HFD  HFD+EGCG  Control  HFD  HFD+EGCG  43kDa  55kDa  33kDa  180kDa  130kDa  100kDa  72kDa  55kDa  43kDa  33kDa  25kDa  17kDa  β-actin  55kDa  33kDa  43kDa  180kDa  130kDa  100kDa  72kDa  55kDa  43kDa  33kDa  25kDa  17kDa  **Repeat 5**  ‘ |
| Fig 8a p-IRS1(Ser307)/IRS1   | Raw data of Fig 8a p-IRS1(Ser307)/IRS1  180kDa  130kDa  100kDa  72kDa  55kDa  43kDa  33kDa  25kDa  17kDa  8kDa  p-IRS1  Control  HFD  HFD+EGCG  Control  HFD  HFD+EGCG  Control  HFD  HFD+EGCG  Control  HFD  HFD+EGCG  180kDa  IRS1  130kDa  β-actin  43kDa  33kDa  180kDa  130kDa  100kDa  72kDa  55kDa  43kDa  33kDa  25kDa  17kDa  p-IRS1  Control  HFD  HFD+EGCG  Control  HFD  HFD+EGCG  180kDa  IRS1  130kDa  100kDa  43kDa  33kDa  β-actin  **Repeat 1**  **Repeat 2**  **Repeat 3**  180kDa  130kDa  100kDa  72kDa  55kDa  43kDa  33kDa  25kDa  17kDa  180kDa  130kDa  100kDa  72kDa  55kDa  43kDa  33kDa  25kDa  17kDa  Control  HFD  HFD+EGCG  Control  HFD  HFD+EGCG  180kDa  p-IRS1  Control  HFD  HFD+EGCG  Control  HFD  HFD+EGCG  130kDa  IRS1  43kDa  33kDa  β-actin  p-IRS1  IRS1  β-actin  **Repeat 4**  **Repeat 5**  **Note: Due to excessive exposure of the final p-IRS1/IRS1 dark-background image, resulting in poor β-actin, p-IRS1, and IRS1 visualization, the entire membrane was additionally displayed with its developed image on a white background.** |
| Fig 8a p-Akt/Akt    180kDa  130kDa  100kDa  72kDa  55kDa  43kDa  33kDa  25kDa  17kDa  Control  HFD  HFD+EGCG  Control  HFD  HFD+EGCG  72kDa  55kDa  p-Akt  Control  HFD  HFD+EGCG  Control  HFD  HFD+EGCG  72kDa  55kDa  Akt  43kDa  33kDa  β-actin  Control  HFD  HFD+EGCG  72kDa  55kDa  p-Akt  Control  HFD  HFD+EGCG  72kDa  55kDa  Akt  43kDa  33kDa  β-actin  180kDa  130kDa  100kDa  72kDa  55kDa  43kDa  33kDa  25kDa  17kDa  8kDa  **Repeat 1**  **Repeat 2**  **Repeat 3** | Raw data of Fig 8a p-Akt/Akt  180kDa  130kDa  100kDa  72kDa  55kDa  43kDa  33kDa  25kDa  17kDa  180kDa  130kDa  100kDa  72kDa  55kDa  43kDa  33kDa  25kDa  17kDa  180kDa  130kDa  100kDa  72kDa  55kDa  43kDa  33kDa  25kDa  17kDa  Control  HFD  HFD+EGCG  Control  HFD  HFD+EGCG  72kDa  55kDa  p-Akt  Control  HFD  HFD+EGCG  Control  HFD  HFD+EGCG  72kDa  55kDa  Akt  43kDa  33kDa  β-actin  **Repeat 4**  **Repeat 5**  **Note: Akt is stripped down from p-Akt.** |
| Fig 9a Beclin1/β-actin | Raw data of Fig 9a Beclin1/β-actin  180kDa  130kDa  100kDa  72kDa  55kDa  43kDa  33kDa  25kDa  17kDa  Beclin1  72kDa  55kDa  43kDa  33kDa  β-actin  Control  HFD  HFD+EGCG  Control  HFD  HFD+EGCG  180kDa  130kDa  100kDa  72kDa  55kDa  43kDa  33kDa  25kDa  17kDa  Control  HFD  HFD+EGCG  Beclin1  Control  HFD  HFD+EGCG  72kDa  55kDa  43kDa  33kDa  β-actin  180kDa  130kDa  100kDa  72kDa  55kDa  43kDa  33kDa  25kDa  17kDa  Beclin1  72kDa  55kDa  43kDa  33kDa  β-actin  Control  HFD  HFD+EGCG  Control  HFD  HFD+EGCG  **Repeat 1**  **Repeat 2**  **Repeat 3**  180kDa  130kDa  100kDa  72kDa  55kDa  43kDa  33kDa  25kDa  17kDa  Control  HFD  HFD+EGCG  72kDa  55kDa  Beclin1  Control  HFD  HFD+EGCG  43kDa  33kDa  β-actin  180kDa  130kDa  100kDa  72kDa  55kDa  43kDa  33kDa  25kDa  17kDa  Beclin1  β-actin  180kDa  130kDa  100kDa  72kDa  55kDa  43kDa  33kDa  25kDa  180kDa  130kDa  100kDa  72kDa  55kDa  43kDa  33kDa  25kDa  Control  HFD  HFD+EGCG  72kDa  55kDa  Beclin1  Control  HFD  HFD+EGCG  43kDa  33kDa  β-actin  **Repeat 4**  **Repeat 5**  **Note: Due to excessive exposure of the final Beclin1 dark-field image, resulting in poor actin visualization, the entire membrane was subsequently stained against a white background.** |
| Fig 9a p62/β-actin | Raw data of Fig 9a p62/β-actin  180kDa  130kDa  100kDa  72kDa  55kDa  43kDa  33kDa  25kDa  17kDa  p62  62  72kDa  55kDa  43kDa  33kDa  β-actin  Control  HFD  HFD+EGCG  Control  HFD  HFD+EGCG  Control  HFD  HFD+EGCG  Control  HFD  HFD+EGCG  180kDa  130kDa  100kDa  72kDa  55kDa  43kDa  33kDa  25kDa  17kDa  p62  62  72kDa  55kDa  43kDa  33kDa  β-actin  Control  HFD  HFD+EGCG  Control  HFD  HFD+EGCG  **Repeat 1**  **Repeat 2**  **Repeat 3**  180kDa  130kDa  100kDa  72kDa  55kDa  43kDa  33kDa  25kDa  180kDa  130kDa  100kDa  72kDa  55kDa  43kDa  33kDa  25kDa  Control  HFD  HFD+EGCG  72kDa  55kDa  p62  Control  HFD  HFD+EGCG  43kDa  33kDa  β-actin  **Repeat 4**  300KDa  250KDa  200KDa  160KDa  130KDa  100KDa  72KDa  45KDa  25KDa  Control  HFD  HFD+EGCG  72kDa  p62  Control  HFD  HFD+EGCG  45kDa  β-actin  300KDa  250KDa  200KDa  160KDa  130KDa  100KDa  72KDa  45KDa  25KDa  **Repeat 5** |
| Fig 9a LC3II/LC3I | Raw data of Fig 9a LC3II/LC3I  180kDa  130kDa  100kDa  72kDa  55kDa  43kDa  33kDa  25kDa  17kDa  8kDa  LC3I  17kDa  8kDa  43kDa  33kDa  β-actin  LC3II  Control  HFD  HFD+EGCG  Control  HFD  HFD+EGCG  Control  HFD  HFD+EGCG  Control  HFD  HFD+EGCG  Control  HFD  HFD+EGCG  Control  HFD  HFD+EGCG  **Repeat 1**  **Repeat 2**  **Repeat 3**  180kDa  130kDa  100kDa  72kDa  55kDa  43kDa  33kDa  25kDa  17kDa  180kDa  130kDa  100kDa  72kDa  55kDa  43kDa  33kDa  25kDa  17kDa  Control  HFD  HFD+EGCG  43kDa  33kDa  β-actin  Control  HFD  HFD+EGCG  17kDa  LC3I  LC3II  180kDa  130kDa  100kDa  72kDa  55kDa  43kDa  33kDa  25kDa  17kDa  180kDa  130kDa  100kDa  72kDa  55kDa  43kDa  33kDa  25kDa  17kDa  Control  HFD  HFD+EGCG  43kDa  33kDa  β-actin  Control  HFD  HFD+EGCG  17kDa  LC3I  LC3II  **Repeat 4**  **Repeat 5** |
| Fig 10a p-AMPKα/AMPKα | Raw data of Fig 10a p-AMPKα/AMPKα  300kDa  250kDa  200kDa  160kDa  130kDa  100kDa  72kDa  45kDa  25kDa  300kDa  250kDa  200kDa  160kDa  130kDa  100kDa  72kDa  45kDa  25kDa  AMPKα  72kDa  45kDa  β-actin  p-AMPKα  72kDa  45kDa  β-actin  p-AMPKα  Cut out the marker  Control  HFD  HFD+EGCG  Control  HFD  HFD+EGCG  72kDa  55kDa  AMPKα  45kDa  β-actin  45kDa  β-actin  Control  HFD  HFD+EGCG  Control  HFD  HFD+EGCG  Cut out the marker  180kDa  130kDa  100kDa  72kDa  55kDa  43kDa  33kDa  25kDa  180kDa  130kDa  100kDa  72kDa  55kDa  43kDa  33kDa  25kDa  **Repeat 1**  **Repeat 2**    p-AMPKα  72kDa  55kDa  AMPKα  45kDa  β-actin  p-AMPKα  45kDa  β-actin  Control  HFD  HFD+EGCG  Control  HFD  HFD+EGCG  72kDa  55kDa  p-AMPKα  Cut out the marker  72kDa  55kDa  180kDa  130kDa  100kDa  72kDa  55kDa  43kDa  33kDa  25kDa  180kDa  130kDa  100kDa  72kDa  55kDa  43kDa  33kDa  25kDa  17kDa  **Repeat 3**  **Note: AMPKα has a low level of phosphorylated proteins and marker affects its exposure. Therefore the original strip was re-exposed with the marker clipped to obtain a clearer band of the target protein. The original bands corresponded one-to-one with the bands after subtracting the marker.**  180kDa  130kDa  100kDa  72kDa  55kDa  43kDa  33kDa  25kDa  17kDa  180kDa  130kDa  100kDa  72kDa  55kDa  43kDa  33kDa  25kDa  17kDa  72kDa  55kDa  AMPKα  180kDa  130kDa  100kDa  72kDa  55kDa  43kDa  33kDa  25kDa  17kDa  Control  HFD  HFD+EGCG  72kDa  55kDa  p-AMPKα  Control  HFD  HFD+EGCG  43kDa  33kDa  β-actin  **Repeat 4**  180kDa  130kDa  100kDa  72kDa  55kDa  43kDa  33kDa  25kDa  17kDa  180kDa  130kDa  100kDa  72kDa  55kDa  43kDa  33kDa  25kDa  17kDa  180kDa  130kDa  100kDa  72kDa  55kDa  43kDa  33kDa  25kDa  17kDa  72kDa  55kDa  AMPKα  Control  HFD  HFD+EGCG  72kDa  55kDa  p-AMPKα  Control  HFD  HFD+EGCG  43kDa  33kDa  β-actin  **Repeat 5**  **Note: AMPKα is stripped down from p-AMPKα.** |
| Fig 10a p-mTOR/mTOR | Raw data of Fig 10a p-mTOR/mTOR  300kDa  250kDa  200kDa  160kDa  130kDa  100kDa  72kDa  45kDa  300kDa  250kDa  200kDa  160kDa  130kDa  100kDa  72kDa  45kDa  mTOR  300kDa  45kDa  β-actin  p-mTOR  300kDa  45kDa  β-actin  Control  HFD  HFD+EGCG  250kDa  200kDa  250kDa  200kDa  Control  HFD  HFD+EGCG  **Repeat 1**  180kDa  130kDa  100kDa  72kDa  55kDa  43kDa  33kDa  25kDa  17kDa  180kDa  130kDa  100kDa  72kDa  55kDa  43kDa  33kDa  25kDa  17kDa  mTOR  180kDa  43kDa  β-actin  p-mTOR  180kDa  43kDa  β-actin  p-mTOR  Cut out the marker  33kDa  33kDa  Control  HFD  HFD+EGCG  Control  HFD  HFD+EGCG  Control  HFD  HFD+EGCG  Control  HFD  HFD+EGCG  **Repeat 2**  **Repeat 3**  **Note: mTOR has a low level of phosphorylated proteins and marker affects its exposure. Therefore the original strip was re-exposed with the marker clipped to obtain a clearer band of the target protein. The original bands corresponded one-to-one with the bands after subtracting the marker.**  300KDa  250KDa  200KDa  160KDa  130KDa  100KDa  72KDa  45KDa  25KDa  300KDa  250KDa  200KDa  160KDa  130KDa  100KDa  72KDa  45KDa  25KDa  300KDa  250KDa  200KDa  160KDa  130KDa  100KDa  72KDa  45KDa  25KDa  mTOR  300kDa  250kDa  200kDa  160kDa  Control  HFD  HFD+EGCG  Control  HFD  HFD+EGCG  45kDa  β-actin  p-mTOR  300kDa  250kDa  200kDa  160kDa  Control  HFD  HFD+EGCG  Control  HFD  HFD+EGCG  **Repeat 4**  **Repeat 5**  **Note: mTOR is stripped down from p-mTOR.** |
| Fig 10a p-ULK1/ULK1 | Raw data of Fig 10a p-ULK1/ULK1  180kDa  130kDa  100kDa  72kDa  55kDa  43kDa  33kDa  25kDa  17kDa  180kDa  130kDa  100kDa  72kDa  55kDa  43kDa  33kDa  25kDa  17kDa  ULK1  110kDa  43kDa  β-actin  p-ULK1  100kDa  43kDa  β-actin  p-ULK1  Cut out the marker  33kDa  33kDa  Control  HFD  HFD+EGCG  130kDa  Control  HFD  HFD+EGCG  130kDa  180kDa  130kDa  100kDa  72kDa  55kDa  43kDa  33kDa  25kDa  17kDa  180kDa  130kDa  100kDa  72kDa  55kDa  43kDa  33kDa  25kDa  17kDa  ULK1  110kDa  43kDa  β-actin  p-ULK1  100kDa  43kDa  β-actin  p-ULK1  Cut out the marker  33kDa  33kDa  Control  HFD  HFD+EGCG  130kDa  130kDa  Control  HFD  HFD+EGCG  **Repeat 1**  **Repeat 2**  **Repeat 3**  180kDa  130kDa  100kDa  72kDa  55kDa  43kDa  33kDa  25kDa  17kDa  180kDa  130kDa  100kDa  72kDa  55kDa  43kDa  33kDa  25kDa  17kDa  ULK1  110kDa  43kDa  β-actin  p-ULK1  100kDa  43kDa  β-actin  p-ULK1  Cut out the marker  33kDa  33kDa  130kDa  Control  HFD  HFD+EGCG  Control  HFD  HFD+EGCG  130kDa  **Note: ULK1 has a low level of phosphorylated proteins and marker affects its exposure. Therefore the original strip was re-exposed with the marker clipped to obtain a clearer band of the target protein. The original bands corresponded one-to-one with the bands after subtracting the marker.**  180kDa  130kDa  100kDa  72kDa  55kDa  43kDa  33kDa  25kDa  17kDa  180kDa  130kDa  100kDa  72kDa  55kDa  43kDa  33kDa  25kDa  17kDa  43kDa  33Da  β-actin  Control  HFD  HFD+EGCG  130kDa  p-ULK1  Control  HFD  HFD+EGCG  110kDa  ULK1  Control  HFD  HFD+EGCG  Control  HFD  HFD+EGCG  **Repeat 4**  **Repeat 5** |
| Fig 10a SIRT1/β-actin | Raw data of Fig 10a SIRT1/β-actin  180kDa  130kDa  100kDa  72kDa  55kDa  43kDa  33kDa  25kDa  17kDa  180kDa  130kDa  100kDa  72kDa  55kDa  43kDa  33kDa  25kDa  17kDa  SIRT1  130kDa  100kDa  43kDa  33kDa  β-actin  Control  HFD  HFD+EGCG  Control  HFD  HFD+EGCG  Control  HFD  HFD+EGCG  Control  HFD  HFD+EGCG  SIRT1  130kDa  100kDa  43kDa  33kDa  β-actin  Control  HFD  HFD+EGCG  Control  HFD  HFD+EGCG  **Repeat 1**  **Repeat 2**  **Repeat 3**  180kDa  130kDa  100kDa  72kDa  55kDa  43kDa  33kDa  25kDa  17kDa  Control  HFD  HFD+EGCG  130kDa  100kDa  SIRT1  Control  HFD  HFD+EGCG  43kDa  33kDa  β-actin  180kDa  130kDa  100kDa  72kDa  55kDa  43kDa  33kDa  25kDa  17kDa  SIRT1  β-actin  **Repeat 4**  **Note: Due to excessive exposure of the SIRT1 dark-field image, resulting in poor β-actin and SIRT1 visualization, an additional strip of the entire film without the black background overlay is displayed.**  180kDa  130kDa  100kDa  72kDa  55kDa  43kDa  33kDa  25kDa  17kDa  180kDa  130kDa  100kDa  72kDa  55kDa  43kDa  33kDa  25kDa  17kDa  Control  HFD  HFD+EGCG  130kDa  100kDa  SIRT1  Control  HFD  HFD+EGCG  43kDa  33kDa  β-actin  **Repeat 5** |

**Note:**

**To probe multiple proteins simultaneously, membranes were cut into horizontal strips according to molecular weight. These strips can be directly traced back to the original full-length membranes.For the experiments in Repeats 4 and 5, full-membrane visualization was performed to further ensure unambiguous correspondence. The raw data is organized to show the complete experimental workflow: the left column shows the original membranes with pre-stained molecular weight markers used to guide membrane cutting; the center column displays the raw chemiluminescent exposures; and the far-right column presents the synthesized luminescent bands with black background to confirm band identity. The green box indicates the representative proteins used in the manuscript, while the blue box illustrates additional Repeats of representative proteins used for data analysis.**
